# Multiscale Computational and Artificial Intelligence Models of Linear and Nonlinear Composites: A Review

**DOI:** 10.1002/smsc.202300185

**Published:** 2024-03-19

**Authors:** Mohit Agarwal, Parameshwaran Pasupathy, Xuehai Wu, Stephen S. Recchia, Assimina A. Pelegri

**Affiliations:** ^1^ Mechanical and Aerospace Engineering Rutgers University‐New Brunswick Piscataway NJ 08854 USA; ^2^ Analysis Materials and Prototyping Directorate DEVCOM Armaments Center Military Base 213 NJ‐15 Wharton 07806 NJ USA

**Keywords:** composites, digital twins, finite‐element analysis, machine learning, multi‐scale modeling, non‐linear constitutive models, soft tissue

## Abstract

Herein, state‐of‐the‐art multiscale modeling methods have been described. This research includes notable molecular, micro‐, meso‐, and macroscale models for hard (polymer, metal, yarn, fiber, fiber‐reinforced polymer, and polymer matrix composites) and soft (biological tissues such as brain white matter [BWM]) composite materials. These numerical models vary from molecular dynamics simulations to finite‐element (FE) analyses and machine learning/deep learning surrogate models. Constitutive material models are summarized, such as viscoelastic hyperelastic, and emerging models like fractional viscoelastic. Key challenges such as meshing, data variability and material nonlinearity‐driven uncertainty, limitations in terms of computational resources availability, model fidelity, and repeatability are outlined with state‐of‐the‐art models. Latest advancements in FE modeling involving meshless methods, hybrid ML and FE models, and nonlinear constitutive material (linear and nonlinear) models aim to provide readers with a clear outlook on futuristic trends in composite multiscale modeling research and development. The data‐driven models presented here are of varying length and time scales, developed using advanced mathematical, numerical, and huge volumes of experimental results as data for digital models. An in‐depth discussion on data‐driven models would provide researchers with the necessary tools to build real‐time composite structure monitoring and lifecycle prediction models.

## Graphical abstract

This research reviews state‐of‐the‐art and latest advances in multiscale modeling methods spanning molecular, micro‐, meso, and macroscale models for hard and soft composites. These models range from molecular dynamics simulations, finite‐element analyses to machine learning/deep learning surrogate models simulating nonlinear constitutive material physics to provide readers with a clear outlook on futuristic trends and tools for composites multiscale modeling.

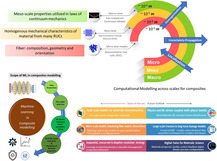

## 1 Introduction

### 1.1 Motivation for Multiscale Modeling

#### 1.1.1 Need for Simulation in Composites

Multiscale (MS) modeling (MSM) of composite materials is a key tool in depicting emergent structural material property functions^[^ ^1^ ^]^ involving multiple physics and across multiple scales. This review article broadly considers partial differential equations (PDE)‐based MS models, which describe composite materials that are spatially heterogeneous and with regionally varying material property functional fields. Depending on the scale of interest, PDE models of composites can be studied using molecular methods (MM), finite difference (FD), or finite‐element (FE) methods which yield approximate solutions. Most functional composites can broadly be classified as having an elastic, viscoelastic, or elastoplastic constitutive behavior. However, bio‐/soft tissue composite materials are more complicated to model. Exhaustively formulated MS models can capture soft tissue responses’ observed mechanics to biological, chemical, or mechanical cues.^[^ ^2^ ^]^ Unlike conventional hard composites, biological soft tissue composites show various attributes such as force generation, active contractions, rearrangement in tissue architecture due to surrounding biological, chemical, or electrical fields, and size variations. Increasing computing power has enabled modern MSM techniques to incorporate broader underlying principles such as kinetics, mass balance, and thermodynamic laws, yielding a more accurate representation of the modeled systems. In recent years, MS models have garnered much interest from researchers because of their effectiveness in depicting composites over a wide range of length scales and mechanistic behavior, such as material characteristics, material degradation, damage, and other complex phenomena (see Section 2.3). In this article, the authors have attempted to review several MS frameworks for soft and hard composite materials. The accuracy‐cost trade‐off and research impact of these models have also been explored. This review intends to provide a consolidated single‐snapshot review of state‐of‐the‐art MS models, both soft and hard composites. The authors believe this is a first‐of‐a‐kind review effort that will enable researchers to gauge the computational landscape for both soft and hard composite domains. Furthermore, discussions on various MS FEM and machine learning (ML)/deep learning (DL) models would encourage the transfer of learning and adaptation of computational models across hard and soft composite spaces in years to come.

#### 1.1.2 Hard Composites

Fiber‐reinforced polymer (FRP) composites are heterogeneous and multiphasic engineered materials in which a polymer matrix is reinforced with artificial, natural fiber, or other reinforcing material. The matrix binds the reinforcement together and transfers the loads between the fibers. It also protects the fibers from any form of environmental or external damage. The fibers/reinforcements provide strength, rigidity, and stiffness to the matrix and obstruct crack propagation and fracture in the structure.^[^ ^3^, ^4^ ^]^ FRP composite materials could also contain other fillers, additives, core materials, or surface finishes designed to enhance the appearance and product performance via more accessible material manufacturing processes. MSM has enabled material scientists to simulate several blend compositions and investigate unique material properties, thus yielding advanced material synthesis. For instance, fiber reinforcements in composites can be unidirectional (UD) or bidirectional. MS FEM can help estimate stiffness (isotropic/anisotropic/orthotropic^[^ ^4^ ^]^) and strength properties along or transverse to the fiber direction. FEM models could help understand damage initiation in the composite matrix, fiber, or interfaces when exposed to extreme environmental (e.g., temperature and pressure) or loading (static/dynamic) conditions.^[^ ^5^ ^]^ Various damage initiation mechanisms, such as transverse matrix fracture, fiber–matrix interface detachment, fiber rupture, and layer delamination,^[^ ^6^ ^]^ have high constancy simulation scope. The accuracy of MS models depends immensely on how particularities related to composite materials, such as constitutive law, modeling, and failure criteria for each composition, are defined, along with an appropriate choice of meshing and element types used to model the FEM objects. Moreover, these MS FEMs often require use of nonlinear (higher‐order) discretized elements and polynomial order (constitutive material models) in numerical solvers, which presents opportunities of further improvement in computational research to closely approximate these nonlinear characteristics as much as possible. Hence, this article reviews such nonlinear modeling efforts for composites in greater detail.

#### 1.1.3 Soft Composites

Tissue mechanics is the study of the mechanical properties of soft biological tissues to mechanical loads and deformation. It combines principles from diverse fields such as materials science, biology, physiology, and applied mathematics. MS computational modeling in soft tissues further subsumes computational science, engineering, and interfacial mechanics. MSM in soft composites spans a wide range of simulation scales, from molecular dynamics (MD) studies for modeling microtubules (MTs) in the brain to organ‐ and tissue‐level continuum models. Tissue mechanics is important for understanding tissue response to various physiological and pathological conditions, such as injury, disease, aging, and regeneration.

Many tissues such as muscles, skin, cartilage, and brain white and gray matter are made of fibers distributed inside a matrix. While experimental studies of tissues such as bone and cartilage have a large amount of data, other soft tissues, such as the brain, have a dearth of direct experimental data for mechanical characterization. Numerous factors contribute to the uncertainty and dearth of knowledge regarding the mechanical characteristics of brain function. First, the brain is entirely enclosed and is hard to probe. Also, depending on the brain region, the distribution of fibers varies from highly directional to randomly oriented, resulting in a wide range of anisotropy. Techniques to study the brain, such as ex vivo experimental techniques, involve a range of strains under relaxation/creep and tissue indentation to characterize tissue‐level mechanical response. Different soft biological materials, such as hydrogels, elastomers, and biological fibers, such as collagen, elastin, and fibrin, are then used to match the experimental data. In vivo methods employ low‐frequency harmonic microstrains using magnetic resonance imaging (MRI). MSM offers a promising path for developing a deeper understanding of soft composite tissue mechanics. Methods such as finite‐element analysis (FEA), MD, and continuum mechanics can be useful in studying a wide range of soft tissues, from individual proteins and collagen fibers to entire organs.

#### 1.1.4 Key Benefits of Analytical/Numerical Modeling

Micromechanical models that account for material nonlinearity in composites could help predict the initiation and propagation of the damage.^[^ ^7^ ^]^ Zhang et al.^[^ ^8^ ^]^ summarized several methods, such as the self‐consistent theory, finite‐element method (FEM), variational calculus method, bridging model, cell structural method, and the secant and tangent modulus methods based on the equivalent inclusion theory, to name a few to study the micromechanical nonlinear material properties of continuous fiber‐reinforced composites over the years. Among these methods, FEM has emerged as a widely deployed tool in predicting critical failure behavior in both soft and hard composite structures.^[^ ^9^ ^]^

FEM‐based damage models incorporating inherent length scale factors, material performance, maintenance period, and structural lifetime predictors^[^ ^7^, ^10^, ^11^ ^]^ can yield accurate failure initiation propagation modes and thereby predict damage evolution. They are deployable to protect material integrity, repair structure, make quantitative material properties predictions, and rational composite design.^[^ ^12^ ^]^ Micromechanical‐scale FEM for composite materials also aids in predicting elastic–plastic stress–strain curves, which can be used instead of experimental testing of composites to establish macromechanical properties.^[^ ^13^ ^]^ Modern general‐purpose explicit FEM codes such as Sierra/Solid Mechanics (Sierra/SM) use non‐linear Lagrangian solid mechanics principles to model the response of a wide range of soft and hard composite materials when subjected to mechanical loading. Such codes provide simulation engineers with extensive constitutive models (CMs), scalability for massively parallel computing, robust contact algorithms, and various techniques for modeling failure in composite material structures. For instance, the continuum damage mechanics (CDM) model first proposed by Kachanov^[^ ^14^, ^15^ ^]^ has numerous applications in studying microcracking phenomena in composite materials. CDM uses an energy‐based constitutive relationship that includes the effect of damage for composite laminate structures via a homogenized damage field vector. Combining intralaminar and interlaminar, 3D FEM models have also been deployed to predict the crush response of composite materials‐built energy‐absorbing structures.^[^ ^14^ ^]^

#### 1.1.5 Scope of Machine Learning in Composite Modeling

Materials research has evolved steadily over recent decades, increasingly incorporating coupled methods that improve the reliability of computational predictions through experimental validation.^[^ ^15^ ^]^ These models include FEM, phase‐field modeling (PFM), and MD simulations that are complex and computationally intensive. As a result, machine learning (ML) methods are being viewed as a promising alternative for material design synthesis. ML models reduce the need for intensive experimental or computational models in predicting resultant composite behavior. ML models find various applications in composite materials research, ranging from property prediction, optimization, feature identification, uncertainty quantification, reliability, and sensitivity analysis.^[^ ^15^ ^]^ ML models could be both forward or inverse models.^[^ ^16^ ^]^ They have extended the capability of traditional numerical and experimental models.^[^ ^15^ ^]^

**Figure 1:**
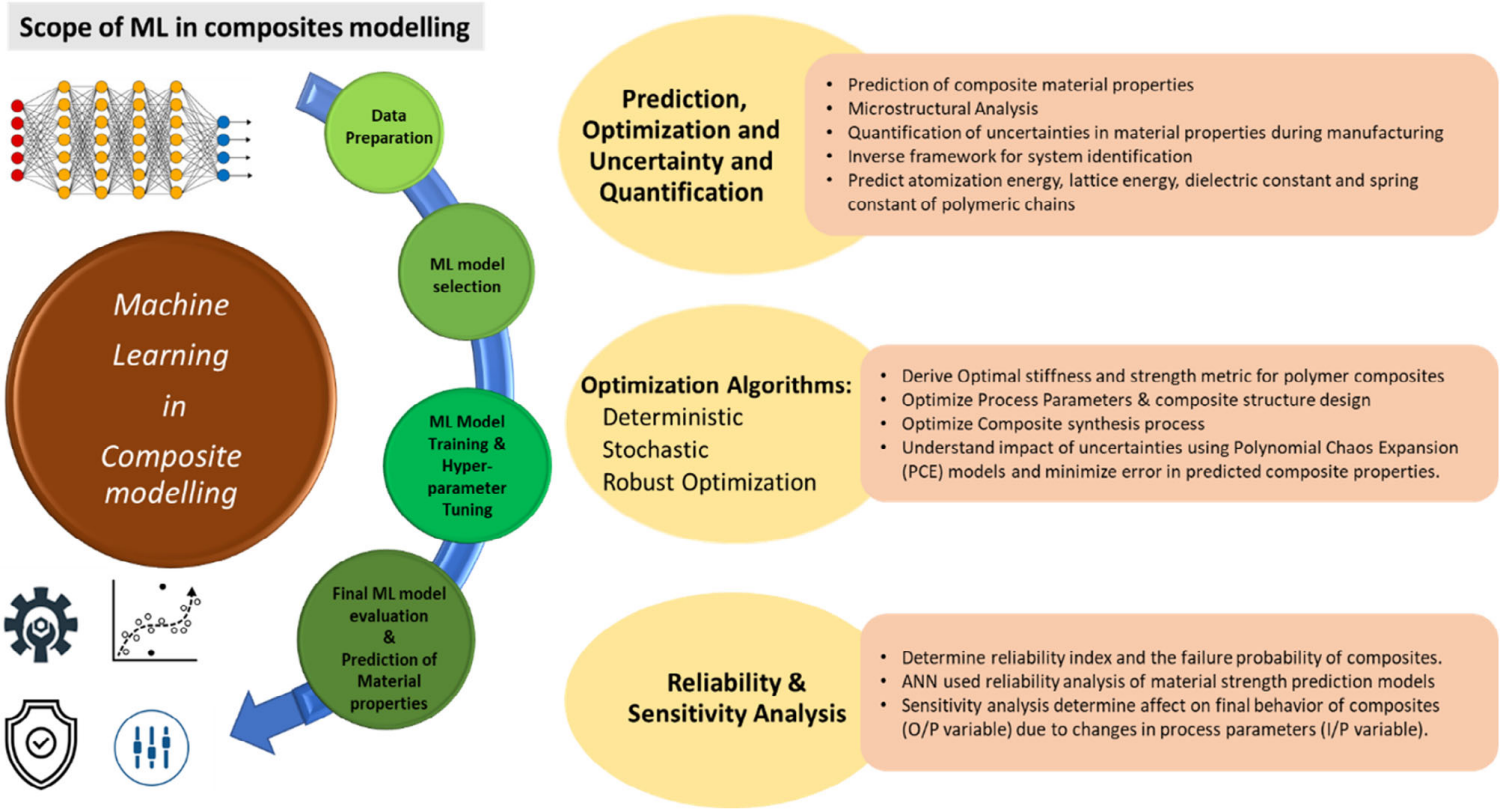
Scope of ML in numeric modeling of composites.

For composite material modeling, ML models help overcome the high computational cost of numerical simulations such as 3D FEA.^[^ ^17^ ^]^ The repeatability of ML model results has encouraged material scientists to deploy them in designing and developing new composite materials with superior properties.^[^ ^18^ ^]^ Numerous ML applications in polymer composite and materials research include property prediction (/characterization), novel material discovery, and several other areas such as damage evolution and analysis, lifecycle, interface analysis, phase, and diagrams (see **Figure** **1**).

ML models can be categorized into supervised and unsupervised ML algorithms. In supervised ML, a well‐labeled dataset is made available, and input parameters are mapped to known target properties/output variables to yield predictions for unseen/test data for predictive modeling. On the other hand, unsupervised ML models rely on discovering patterns in the dataset based on clustering and association rules. Supervised ML models are top rated in polymer composites research and are often deployed as predictive models for estimating composite material behavior. Optimization problems using stochastic algorithms can significantly improve efficacy and efficiency by leveraging ML models.^[^ ^19^, ^20^, ^21^, ^22^ ^]^ Some of the popularly applied ML models include artificial neural networks (ANNs), conventional NN (CNNs), recurrent neural networks, ANN + Adaptive neurofuzzy inference systems, ANN + genetic algorithm (GA), backpropagation neural network‐GA, etc. Neural networks are robust and efficient in handling copious amounts of data and using nonlinear mapping functions. Some of the pioneering works for introducing ML to composite modeling are advanced composite stress–strain sensing,^[^ ^23^ ^]^ tribological characteristics of polymer composites,^[^ ^24^ ^]^ wear damage behavior modeling of composites as a function of composition and test condition,^[^ ^25^, ^26^ ^]^ and determining material properties concerning computational material design.^[^ ^27^, ^28^ ^]^ Sehyan et al. applied a feed‐forward ANN to predict the compressive strength of glass‐reinforced polyester composites and understood the influence of applied manufacturing methods.^[^ ^29^ ^]^ Farhangdoust et al. located defects in connection interface of the polymer composite^[^ ^30^, ^31^ ^]^ and ANN and neurofuzzy inference systems to predict the mechanical behavior of glass‐reinforced plastic composites (GRPC) impact strength,^[^ ^32^ ^]^ yield strength, and fatigue response of composite materials.^[^ ^33^, ^34^, ^35^, ^36^ ^]^

ML models come with their own set of challenges and limitations. First, they suffer from the curse of dimensionality. However, this can be overcome by trying out specific dimensionality reduction techniques such as principal component analysis (PCA), multicollinearity analysis, regularization, sensitivity analysis, feature ranking, variance filters, independent component analysis, etc.^[^ ^15^ ^]^ ML models also commonly struggle with overfitting and underfitting issues. Bias‐variance tradeoff analysis, regularization, and feature selection can help overcome such challenges and improve ML model accuracy. Another critical challenge with MS ML models is that they must be trained, tested, and validated with MS data.^[^ ^37^ ^]^ However, MS data is hard to obtain, and the scale of data is limited. Noise in the dataset also challenges the ML models’ prediction classification accuracy and increases the learning time.

Furthermore, high‐fidelity simulations such as 3D FEA and complicated DL architecture are expensive.^[^ ^17^ ^]^ This is especially challenging in soft tissue (soft composite) space, where the data is limited and expensive. Moreover, since biological systems are highly nonlinear, the ML/DL approach often fails to capture underlying physics due to sparse data. Even for hard composites, scaling physical parameters across MSMs faces repeatability and feasibility issues during experimentation. Compounding these challenges, computational models frequently do not have real‐world data as true labels when building scalable ML/DL predictive models. DL algorithms lack interpretability to accurately describe the physics between algorithmic modeling and biological structural responses, thus creating additional bottlenecks in deploying these models.

Hybrid ML models (ML + FEM) with sufficient intricacies and manual tuning can solve high‐dimensional problems for generalizing to unseen data. Hybrid computational models, multifidelity ML models, or hybrid ML models combining multiple ML algorithms have the potential to outperform individual ML methods used to predict composite properties. Adaptive ML has enormous potential for building scale‐bridging models (nano‐ to continuum scale) for developing efficient nanocomposites. Physics‐informed ML is another promising solution for integrating data and mathematical models while addressing the intricacies of mesh generation, inverse problems, and high‐dimensional spaces governed by high‐order differential equations.

### 1.2 Evolution of MSM

Traditionally, material scientists relied on experimental methods to discover and characterize new materials, but advanced computational capabilities have revolutionized the field of material science. Computational models have complemented experimental research in generating realistic simulations for material discovery. The efficient use of computational simulations and experimental mechanics has yielded abundant new opportunities for material discovery. Realistic computational models require understanding morphological and structural evolution schemes, damage/delamination, and nonlinear damage mechanics to predict fatigue and fracture in both soft and hard composites.^[^ ^4^ ^]^

In its infancy, limited by computing power, simulations were done at coarse or continuum scales from which homogenized composite characteristics could be derived and validated experimentally. As technology advanced, scales of MS simulations have broadened from coarse to very fine scale, with the ability to simulate complex dynamical evolution mechanics.^[^ ^38^ ^]^ However, today, computational models span multiple lengths and timescales from continuum to micro‐/molecular levels for predicting composite properties. Recently, multigrid methods have also emerged, allowing material scientists to interlink overall high‐frequency composite structure responses via intercalating transfer operators via homogenization. As computing hardware and programs evolve, MS asymptotic, multiresolution methods, and enrichment schemes continue to flourish.

**Figure 2:**
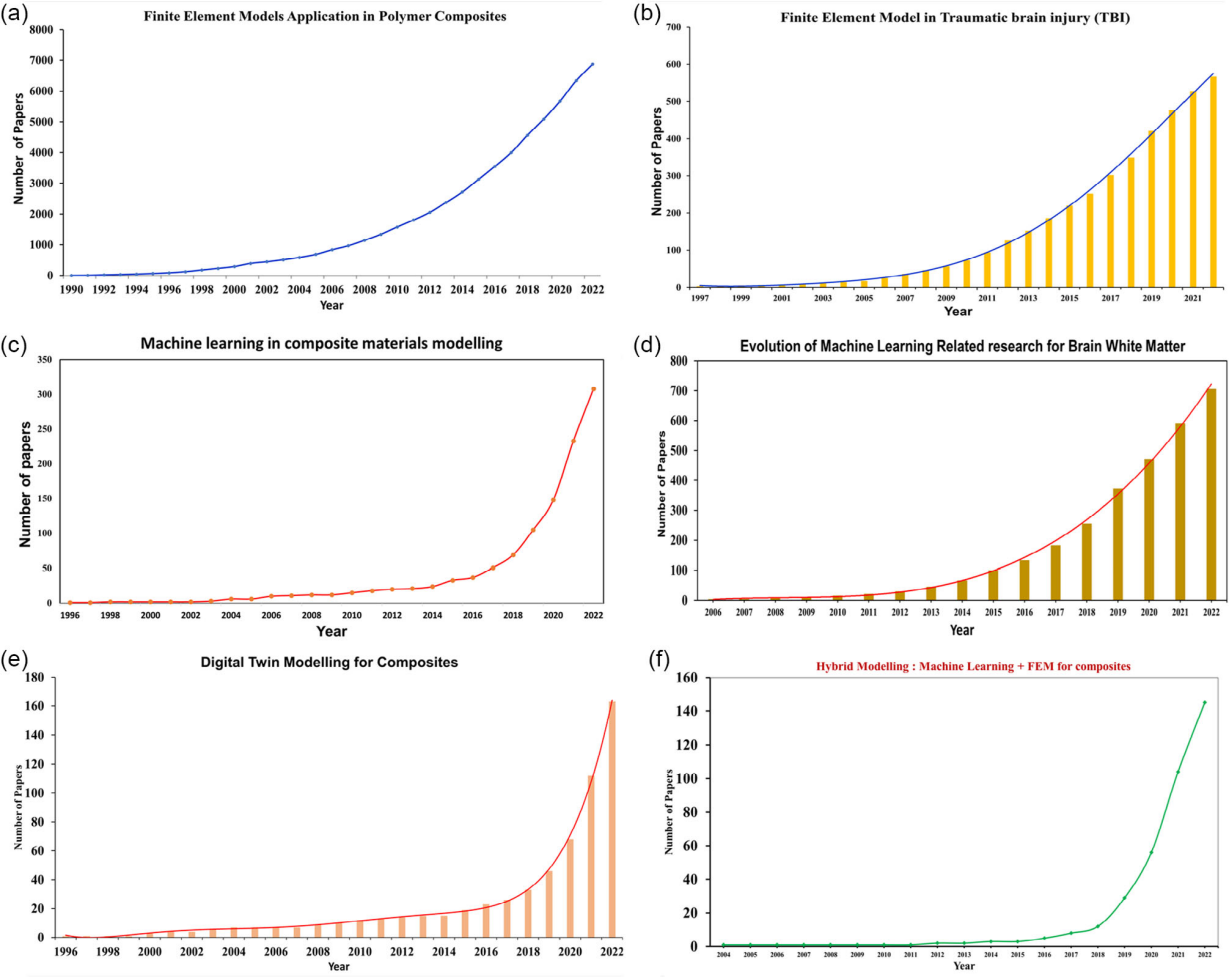
Evolution of MSM shown by the number of plotting publications over the years. a) Publication trends in FEM for polymer composites highlighting exponential growth over the past 25 years. b) The growth trend in adopting FEM for TBIs numerical models. c) ML models in composite materials have leaped tremendously in the past 10 years. d) Given the complexity of the parametric modeling of BWM tissues, ML is becoming an increasingly popular alternative. e) DT models for composites have radically increased in the past 5 years and continue to rise as part of the Industry 4.0 revolution for advanced composite manufacturing. f) Hybrid models combining FEM + ML leverage ROM models to depict complex composites and recently have gained much traction in the composite modeling research community, as seen by publication trend plots.

Since the advent of ML and artificial intelligence (AI) toolkits, data‐driven methods have immensely aided in the development of coarse‐grained or reduced‐order models (ROM)/surrogate models. Hybrid modeling techniques where fine‐scale simulation data are used to develop part‐scale ROM using ML/AI approaches are discussed in detail in the latter sections. As shown from plots in **Figure** **2**, the application of ML models in composite modeling research has increased exponentially over the past decade. Note: this data analysis was conducted in‐house via scrapping and advanced search methods for keywords for different plot categories (Figure 2) in literature databases such as Google Scholar, Science Direct/Elsevier, and Web of Science to obtain the relevant number of published papers on the respective topics. For the trend analysis, data from 1990–2022 was scoped, and the number of articles with these words was counted cumulatively to create aggregation plots. These ML‐driven composite research trends were observed for hard and soft composite materials. ML‐related research for BWM first emerged in the mid‐2000s. However, over the past decade, the research output of ML‐driven traumatic brain injury (TBI) has increased seven times (Figure 2c,d), highlighting the vast scope of stochastic/statistical modeling techniques to characterize soft tissue behavior which is highly indeterministic and challenging to model constitutively. FEMs have significantly reduced project costs, enabled real‐size component and equipment simulations, and simulate complex boundary swiftly and accurately. This is evident from the steep growth of FEM application in polymer composite and soft tissue research, such as modeling of TBI in BWM (see Figure 2a,b).

ML‐driven composite models have been deployed for a host of purposes such as property prediction, novel material discovery, composite damage assessment, phase/constituent distribution diagrams, precision machining, manufacturing process optimization, life span prediction, residual stress estimation, temperature distribution and degree of cure in polymer composites, etc. Newer ML models combine a multifidelity approach by merging low‐fidelity and expensive high‐fidelity input data optimally to achieve relatively high‐accuracy predictions.^[^ ^15^ ^]^ Hybrid modeling by combining ML and FEM makes it possible to develop MS representations of complex polymer composites. Digital twin (DT) for materials science has proliferated in the past decade due to hybrid modeling and edge computing hardware innovations that generate close‐to‐real‐time material property predictions. These technologies continue to gain higher traction, as shown by research throughput trends in Figure 2e,f. These latest modeling approaches have been further detailed in subsequent sections.

### 1.3 Scales of MS

#### 1.3.1 Current State of the Art/Background

Composites are inherently MS, whereby lower‐order microscopic constituent contributes to the resultant macroscopic material.^[^ ^39^ ^]^ Recent efforts in MSM aim to bridge the simulation gaps between scales from molecular to mesoscale levels while working over a broad range of timescales. These approaches have proven extremely useful in establishing the relationship between microstructure behavior and macroscopic properties. By coupling micro‐ and macroscale computational models, accurate material representations can be realized when compared to continuum‐scale simulations alone.

Accuracy and cost requirements tests determine deployment decisions and qualification of MSM approaches. These tests are based on the cost ratio of the multi‐ and microsolver, estimation errors in quantities of interest.^[^ ^40^, ^41^ ^]^ Depending on the bridging method, MS simulation models can be grouped into two major types: hierarchical or concurrent methods.^[^ ^39^, ^42^ ^]^ Based on the information exchange and coupling states, they have been further subcategorized into semiconcurrent and hybrid semiconcurrent^[^ ^43^, ^44^ ^]^ MS models. Hierarchical multistrategy is often labeled as the upscaling methods, information‐passing methods, sequential methods, serial methods, coarse‐graining methods, and computational homogenization‐like methods. In hierarchical modeling, micro‐ and macro‐scale problems are solved sequentially, and the information flow could be in either bottom‐up or top‐down sequence between the length scales.

Typically, in a hierarchical model approach, the material information passes unidirectionally from the micro‐ to macroscale following a bottom‐up coupling, while the spatial distribution information passes in the opposite direction in a top‐down approach.^[^ ^45^ ^]^ A substantial number of hierarchical analyses are bottom‐up. This method's highly complex computational microscopic response plots are idealized and averaged. These precomputed predictions are then used to estimate constitutive parameters in the macroscale simulations.^[^ ^46^, ^47^, ^48^ ^]^ Statistical analysis, homogenization, and other bridging methods are leveraged to link these models spread over disparate scales.^[^ ^49^, ^50^, ^51^, ^52^ ^]^ The hierarchical modeling technique offers a simple and efficient information exchange, leading to widespread application in composite material modeling research.^[^ ^53^, ^54^, ^55^ ^]^ State‐of‐the‐art hierarchical models have proven highly efficient and accurate in predicting averaged macroscopic composite material response. However, hierarchical techniques are unreliable in scenarios involving macroscopic inhomogeneity or nonlinearity. It happens due to macroscopic localization, failure, and instability factors, which result in high response variable gradients and invalidate any periodicity hypothesis of homogenization.^[^ ^55^, ^56^, ^57^ ^]^ The concurrent MSM schema aptly addresses these limitations.

The concurrent MSM technique is also called the resolved‐scale, embedded, integrated, or hand‐shaking method. In this method, micro‐ and macroscale models are computed simultaneously on different subdomains and are intensively coupled “on the fly” via mutual information exchange scheme at the model interface.^[^ ^41^, ^49^ ^]^ This concurrency also enforces compatibility requirements and momentum balances to ensure intersubmodels continuity.^[^ ^56^ ^]^ The bridging in the concurrent modes is achieved numerically, and algorithms at different scales are fused via matching procedures at the individual model interfaces/overlapping regions.^[^ ^50^ ^]^ While the concurrent multiscaling approach is computationally expensive compared to hierarchical models, they outweigh the latter in accuracy by limiting scalability errors encountered in solving macroscale models utilizing microscopic information.^[^ ^40^ ^]^ Thus, concurrent MSM strategies are widely preferred in highly heterogeneous composite material or nonlinear process modeling problems.^[^ ^41^, ^53^, ^57^, ^58^ ^]^ Modern numerical modeling approaches involving collaborative utilization of both multiscaling methods could further optimize MSM efficacies. It can be achieved by leveraging concurrently obtained results about the functional forms of constitutive equations in sequentially executed hierarchical methods. As MSM strategies evolve, composite models with information flowing in bottom‐up, top‐down, or concurrent sequences among micro‐, meso‐ and macro‐scales will be developed for real‐world applications.

#### 1.3.2 Scale: Microscale, Molecular Scale, and Meso‐/Macroscale

Numerical modeling can be categorized into three categories based on the extent and intent of investigation: molecular scale, microscale, and mesoscale/macroscale. As discussed in Section 1.3, molecular scale and microscale models are far too intricate to manage for large composite material structure analysis. Besides, such an approach would involve complex procedures to extract macroscopic properties from exacting microscale setups. MSM combines micro‐ and macromodels and leverages the independent efficacies of individual models.^[^ ^39^ ^]^ The intent is to attain a combined macroscopic–microscopic computational setup that is more efficient than solving slow and highly complex micro‐/molecular‐scale models but also provides accurate composite property information.^[^ ^42^ ^]^ Elliott^[^ ^59^ ^]^ elaboratively describes the evolution, motivation, and forums at the forefront of promoting MSM in their review article. It also explains the dissimilarities in the theoretical model framework across different temporal and spatial (length) scales due to changes in model scope. It also discusses opportunities for model mapping/zooming across molecular, micro‐, and macroscales by hierarchical or concurrent approach. This requires homogenization techniques and modeling anisotropy nonlinearity in hard and soft composites.

**Figure 3:**
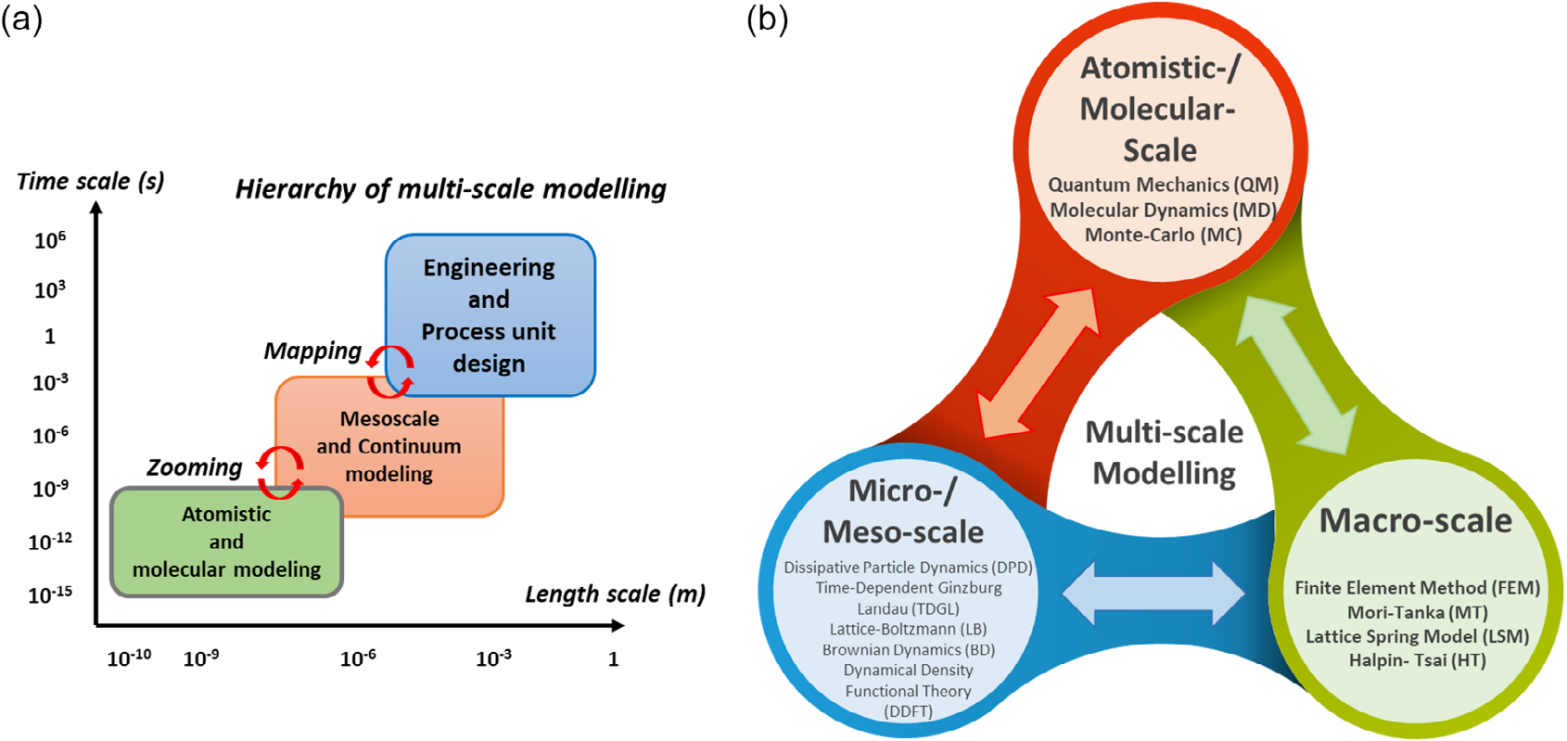
a) Hierarchical representation of MSM (length v/s timescale). Adapted from permission.^[^ ^59^ ^]^ Copyright 2011, ASM International. b) Interrelationships between the atomistic/micro‐/meso‐, and macroscale models and critical techniques within each scale‐based modeling category. Adapted from permission.^[^ ^131^ ^]^ Copyright 2022, Preprints.

Composite material characterization studies can be done from the macro‐ to the molecular scale. At the molecular scale, numerical simulation modeling techniques can help estimate thermodynamic composite synthesis kinetics, structural characteristics, and molecular interactions. Electronic structure calculations and quantum mechanics (QM) methods model their state. At the quantum scale (≈10^−10^ m, ≈10^−12^ s), the formation and rupture of chemical bonds, changes in electrical configurations, and other similar phenomena are typically investigated. At the atomistic scale (≈10^−9^ m, ≈10^−9^–10^−6^ s), forcefield interactions (bonded or nonbonded) are utilized to determine the potential energy of the system.^[^ ^38^ ^]^ MD and Monte Carlo (MC) simulations are popular. Hybrid QM–molecular mechanics (MM) methods, that is, QM–MM models, have also accurately predicted interatomic potentials in atomistic‐scale simulations using linear‐scaling density functional theory (DFT)‐based ONETEP code^[^ ^59^ ^]^ (refer **Figure** **3**). Microscale simulations help understand polymer nanocomposite's (PNC) structural evolution, phase separation, and hydrodynamical behavior. Microscale simulations are often cited as fine, unresolvable, subgrid, or discrete scales.^[^ ^41^ ^]^ In the case of mesoscopic scale models (≈10^−6^ m, ≈10^−6^–10^−3^ s), microscopic particles or beads are used to simulate phenomena at longer lengths and timescales hardly accessible by atomistic methods. Mesoscopic models can be solved with a field or a particle‐based modeling approach. The interactions between the beads are used to characterize the system. Some of the popular models to investigate the mesoscopic structures in polymeric systems include dissipative particle dynamics (DPD), Brownian dynamics (BD), lattice Boltzmann (LB), dynamic density functional theory (DDFT), and time‐dependent Ginzburg‐Landau (TDGL) theory (refer **Table** **1**).

**Table 1:** Summary of popular microstructure‐level models

Microstructure level (micro‐/Mesoscopic scale) Model Name	Key features and model outputs
BD	Random simulation models focused on obtaining diffusion information in composites and polymers. Can work on very minute timescales. It is a subdivision of the microscale method.
DPD	Particle‐based method. DPD simulates interaction potential between substrate and fillers at length and timescales in composite material modeling. It also incorporates flow pattern obeying Navier stokes equation.
LB	Reproduce particle interaction into numerical modeling. Describe various aspects of the polymer's solution characteristics using free energy, thermodynamics, kinetics, and hydrodynamics. LB faces stabilization issues with numerical results.
TDGL	Perform structural analysis at phase separation. Solve nonlinear diffusion. TDGL and cell half‐open method (CDM) are widely used for enthalpic interaction between composite/polymer components and phase separation studies in nanocomposites.
DDFT	Efficiently conforms nonequilibrium density profile in polymer blends/composites modeling by considering the interaction potentials and statistical thermodynamics.

Mesoscopic and macroscopic models are formulated on laws such as continuity (conservation of mass), equilibrium (Newton's second law), the moment of momentum principle (rate of change of angular momentum), conservation of energy (first law of thermodynamics), and the principle of non‐negative entropy (in the second law of thermodynamics) to account for nonisentropic processes such as constant temperature models. These form the roots of the continuum model developed to study stress and strain response in PNCs. At the macroscale, averaged force and energy quantities are considered for numerical simulation.

In macroscopic (≈10^−3^ m, ≈1 s) models, composite systems are treated as a continuous medium and are not considered discrete characteristics of atoms and molecules. Macroscopic systems’ behaviors are governed by constitutive laws often coupled with conservation laws to simulate various material phenomena. Excluding specific continuity separation sites, material property function components are treated continuously. Fundamentally, the macro‐scale heterogeneous material models are replaced with an equivalent homogeneous model. Some of the most widely used macroscale techniques used to simulate composite (soft or hard) systems are the finite difference method (FDM), FEM, and finite volume method (FVM). The latter sections follow an in‐depth discussion of these MS models for soft and hard composite applications, see Figure 3.

## 2 Experimental Section

### 2.1 MSM Techniques

#### 2.1.1 The Objective of MD, FEM, and ML: Prediction of Material Properties and Application in Soft Materials

MS models bridge length scales from micrometers to large composite structures. They have been deployed in myriad applications with advanced materials and structures research. Typical MSM design objectives are as follows: prediction of the macroscopic behavior of complex, layered composites; simulation of complex heterogeneous composite systems to describe large deformation and failure mechanics (primarily time history‐dependent scenarios); mapping continuum‐scale descriptors (deformations or temperature fields) onto smaller‐scale features;^[^ ^49^ ^]^ help formulate material specific constitutive equations; model controlled reaction kinetics of polymer composites and extent of reaction; simulate dispersion phenomena of nanoparticulates, model the range of entropy, enthalpy, and free energy; analyze composite formation dynamics and rate of polymer composites formation to name a few.

MS models have also proved pivotal in advancing soft tissue modeling to simulate several biochemical, biophysical, and biomechanical phenomena^[^ ^60^ ^]^ in various biological systems. Numerical models can also help understand the histology, morphology, and mechanical behavior of complex soft tissues such as brain tissues (gray and WM).^[^ ^61^ ^]^ FEM and ML models have helped simulate and predict the impact of TBIs and also model aging/damage of soft tissues. Fatigue modeling of soft tissues has also garnered much attention in the computational materials research community, driving high‐fidelity numerical solutions that predict tissue decay and damage.^[^ ^62^ ^]^ MS models also help formulate hypotheses about cellular or tissue network‐level biomechanics of soft tissue damage. In an industrial setting, computational soft composite material models (both ML or FEM based) have been integral in designing reliable protective head gears, sports injury biomechanics,^[^ ^63^ ^]^ user safety in vehicle design, human–interface systems design, preventing trauma, planning rehabilitation program, and a host of medical devices design.

Over the years, numerical models of complex soft tissues of several head parts using finite‐element modeling that predict their mechanical response^[^ ^64^, ^65^ ^]^ under various impact scenarios have been developed. Computational research groups, along with their experimental research collaborators,^[^ ^66^, ^67^, ^68^ ^]^ have invested considerable research efforts in computation modeling of BWM (FEM^[^ ^69^, ^70^, ^71^ ^]^ and ML^[^ ^68^ ^]^ based). Over the years, many findings regarding^[^ ^72^ ^]^ the mechanical response of BWM for a variety of impacts (uniaxial to the multiaxial case), TBIs, aging,^[^ ^73^ ^]^ and fatigue load scenarios on the brain tissues^[^ ^74^, ^75^ ^]^ have been put forward. Similarly, researchers from Karami et al. have intensively studied TBI and brain soft tissue composite modeling. Their research has been focused on predicting brain matter properties^[^ ^76^, ^77^ ^]^ using micromechanical to macroscale FEM,^[^ ^78^ ^]^ optimization algorithms,^[^ ^79^ ^]^ data‐based models, hybrid models, inverse numerical models for characterization of brain matter^[^ ^80^ ^]^ and predicting TBIs, blasts,^[^ ^81^ ^]^ and fatigue and dynamic response^[^ ^82^ ^]^ mechanisms under various load cases. Such soft tissue composite computational models and related TBIs’ numerical research have been elaborated on in later sections.

### 2.2 Soft Tissue Composite MSM

#### 2.2.1 Data Acquisition: Experimental Methods

Geometric data for soft tissue MSM could come from actual human medical image data collected by computed tomography (CT), MRI, and other visible human datasets.^[^ ^83^ ^]^ The challenge with data acquisition for MSM is that experimental data is often limited, and the inherent noise in the data can further make it unreliable.^[^ ^37^ ^]^ The MRI‐derived model is widely used as a 3D imaging data source for developing geometric aspects of computational macroscopic and mesoscopic soft tissue models.^[^ ^84^, ^85^ ^]^ CT image data can extract the point data model of the liver by sampling the amount of uniformly distributed particles, thus recreating the same mass and density.^[^ ^86^ ^]^

The mechanical property data of soft tissue, such as nonlinear stress–strain relationship, viscoelasticity, anisotropy, boundary condition, interface contact condition, and other soft tissue parameters, are needed for constructing mechanical models.^[^ ^83^ ^]^ These data are measured from experiments, minimally invasive, or in vivo measurements by sensors and instruments. Image data can also be helpful for the extraction of the mechanical properties of soft tissue. Magnetic resonance elastography (MRE) has been used to characterize tissues like the brain, liver, and muscle in vivo and to study changes in stiffness due to aging, disease, or injury. Atomic force microscopy stress relaxation experiments on corpus callosum, corona radiata, and fornix of the fresh bovine brain are becoming popular means to experimentally determine tissue's mechanical response while considering variations in axon orientation.^[^ ^87^ ^]^

#### 2.2.2 Model Setup: Key Inputs, Boundary Conditions, Outputs

Numerous attempts have been made to characterize soft tissue materials’ anisotropic and nonlinear behavior under different circumstances in vivo and in vitro and under various experimental procedures.^[^ ^77^ ^]^ For example, the literature considers different frequency and amplitude ranges in dynamic shear testing. Furthermore, MS biocomposite structure plays a critical relationship between the stress–strain response of composite and its resulting injury.^[^ ^77^ ^]^ The microstructures of biological tissues such as the arterial wall, skeletal muscle, skin, and brain tissue are bundles of fibrous materials surrounded by an extracellular matrix (ECM). Researchers have used different material modeling approaches to capture the complex mechanical behavior of such tissues. The complexity of the computational simulations of soft tissues depends on the level of the spatiotemporal discretization necessary to capture the finer details and the material models that can effectively describe the subcellular mechanics. In vivo and ex vivo methods used to build mathematical models of tissue typically involve using MRI–MRE data and an optimization technique to fit the data to the computational models.

Sack et al.^[^ ^88^ ^]^ developed subject‐specific biventricular cardiac finite‐element models of a healthy and failing swine heart. Hyperelastic strain energy functions model the heart tissue's active and passive material properties. The Ogden–Holzapfel^[^ ^89^ ^]^ hyperelastic model simulates the anisotropic hyperelastic behavior of tissue containing collagen fibers embedded in an isotropic matrix. Pfensig et al.^[^ ^90^ ^]^ developed finite‐element models of artificial leaflet structures for transcatheter heart valve prostheses using different hyperelastic strain energy functions and fit experimental uniaxial tension and planar shear data to these models. The article estimates that a three‐parameter Ogden model achieved the best fit of the nonlinear stress–strain behavior of the isotropic polymeric prosthesis with the experimental data. Gholipour et al.^[^ ^91^ ^]^ created a biomechanical model of the left and right coronary arteries by extracting the coordinates of the geometry of the arteries from angiogram images. The model used a hyperviscoelastic model using a Mooney–Rivlin hyperelastic material and a Prony series viscoelastic parameters to model the tissue response to time‐dependent artery wall pressure. Estermann et al.^[^ ^92^ ^]^ developed a hyperviscoelastic characterization of bovine and porcine hepatic parenchyma in tension using a Veronda–Westmann^[^ ^93^ ^]^ hyperelastic model and the Prony series parameters under a ramp test.

Specifically focusing on the microstructure of BWM, Meaney^[^ ^94^ ^]^ developed an analytical structural model of the microstructure of highly oriented axons in BWM using different hyperelastic strain energy functions. Zhang et al.^[^ ^95^ ^]^ advanced a computational model combining a MD approach and a neo‐Hookean finite‐element model of the membrane structure of an unmyelinated axon to predict its Young's modulus. Arbogast et al.^[^ ^96^ ^]^ proposed a viscoelastic fiber‐reinforced composite model of axons in ECM in the frequency domain from experiments on porcine optic nerves. Montanino et al.^[^ ^97^ ^]^ introduced a microstructural model of the axon and its substructures under different strain rates to study axonal injury mechanisms. Pan et al.^[^ ^67^ ^]^ (2011) developed a transitional micromechanical model describing the transition of axons from nonaffine‐dominated kinematics at a low stretch to affine kinematics at a high stretch using the Ogden hyperelastic material model. Pan et al.^[^ ^98^ ^]^ (2012) proposed a pseudo‐3D representative volume element (RVE) model of WM tissue using the Ogden hyperelastic material model. Pasupathy et al.^[^ ^69^ ^]^ developed tissue‐level hyperelastic finite‐element models of the BWM by incorporating the tethering effect of oligodendrocytes. Agarwal et al.^[^ ^74^ ^]^ performed a parameterized study of the effect of oligodendrocyte tethering on BWM.

Sullivan et al.^[^ ^99^ ^]^ introduced a triphasic composite model consisting of axons, myelin, and ECM that calculates the homogenized viscoelastic material properties under steady‐state dynamics. Wu et al.^[^ ^75^ ^]^ developed a finite‐element model that merges frequency domain viscoelasticity‐based microscale orthotropic RVEs to a macroscale BWM model, which accounts for local axonal orientation. Nicolas et al.^[^ ^100^ ^]^ performed ex vivo brain experiments using ultrasound shear wave spectroscopy and determined its mechanical parameters by fitting a power‐law model. Pasupathy et al.^[^ ^101^ ^]^ (2022) formulated a fractional viscoelastic model of the microstructure of BWM using the FEM.

#### 2.2.3 Hyperelastic Material Modeling

For a general state of deformation based on a reference configuration, X, the deformation gradient is described as

(1) $$\begin{array}{c} F_{ij}=\frac{\partial x_{i}}{\partial X_{j}} \end{array}$$

The right and left Cauchy–Green tensors are defined as

(2) $$\begin{array}{c} C=F^{T}\cdot F,\;B=F\cdot F^{T} \end{array}$$

The principal invariants of the right Cauchy–Green tensor are

(3a) $$\begin{array}{c} I_{1}=\text{trace}(C)=\lambda_{1}^{2}+\lambda_{2}^{2}+\lambda_{3}^{2} \end{array}$$

(3b) $$\begin{array}{c} I_{2}=\frac{1}{2}({\left(\text{trace}(C)\right)}^{2}-\text{trace}(C^{2}))=\lambda_{1}^{2}\lambda_{2}^{2}+\lambda_{2}^{2}\lambda_{3}^{2}+\lambda_{1}^{2}\lambda_{3}^{2} \end{array}$$

(3c) $$\begin{array}{c} I_{3}=\text{det}(C)=\lambda_{1}^{2}\lambda_{2}^{2}\lambda_{3}^{2} \end{array}$$

where $\lambda_{1}$, $\lambda_{2}$, $\lambda_{3}$ are the principal stretches. The strain energy can be written as a polynomial function of the invariants. A general strain energy formula can be obtained by expanding the function as a power series in terms of the invariants.

(4) $$\begin{array}{c} W\left(I_{1},I_{2},I_{3}\right)=\sum_{i,j,k=0}^{\infty }c_{ijk}{\left(I_{1}-3\right)}^{i}{\left(I_{2}-3\right)}^{j}{\left(I_{3}-1\right)}^{k} \end{array}$$

where ${\text{I}}_{1}=3,{\text{I}}_{2}=3,{\text{I}}_{3}=1,$ and ${\text{c}}_{000}=0$ in the reference configuration. An alternate measure of the strain energy function in terms of the volumetric and deviatoric components can be obtained by introducing $\tilde{C}=J^{-2/3}C,\tilde{I_{1}}=J^{-2/3}I,\tilde{I_{2}}=J^{-4/3}I_{2},$ and $\tilde{I_{3}}=1$. The modified strain energy function is thus

(5) $$\begin{array}{c} W\left(\tilde{I_{1}},\tilde{I_{1}},J\right)=\sum_{i,j=0}^{N}c_{ij}{\left(\tilde{I_{1}}-3\right)}^{i}{\left(\tilde{I_{2}}-3\right)}^{j}+\sum_{p=1}^{M}k_{p}{\left(J-1\right)}^{2p} \end{array}$$

The Cauchy stress tensor is then defined as

(6) $$\begin{array}{c} T=2J^{-1}F\frac{\partial W}{\partial C}F^{T} \end{array}$$

Strain energy functions commonly used in modeling different biological soft tissues are shown in **Table** **2**.

**Table 2:** Summary of strain energy functions for material modeling

Material Model	Strain Energy Function
Fung model^[^ ^212^ ^]^	$W=\frac{c}{2}\left(\exp\left(Q\right)-1\right)+\frac{1}{D}\left(\frac{J^{2}-1}{2}\right)$ where c and D are temperature‐dependent material parameters. $Q=\varepsilon_{\text{ij}}b_{\text{ijkl}}\varepsilon_{\text{kl}}$ . $b_{ijkl}$ It is a dimensionless symmetric fourth‐order tensor of anisotropic material constants. $\varepsilon_{ij}$ These are the components of the Green strain tensor.
Two parameter Mooney–Rivlin model^[^ ^213^, ^214^ ^]^	$W=c_{10}\left(\tilde{I_{1}}-3\right)+c_{01}\left(\tilde{I_{2}}-3\right)+\frac{1}{D}{\left(J-1\right)}^{2}$ $\mu_{0}=2\left(c_{10}+c_{01}\right)$ where $c_{10}$, $c_{01}$, $c_{11,}$ and D are temperature‐dependent material parameters. $\mu_{0}$ is the initial shear modulus. If $c_{01}$ is 0, Mooney–Rivlin model becomes a neo‐Hookean model.
neo‐Hookean model^[^ ^215^ ^]^	$W=c_{10}\left(\tilde{I_{1}}-3\right)+\frac{1}{D}{\left(J-1\right)}^{2}$ $\mu_{0}=2c_{10}$ where $c_{10}$and D are temperature‐dependent material parameters. $\mu_{0}$ is the initial shear modulus.
Ogden model^[^ ^216^ ^]^	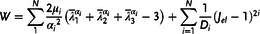 where ${\bar{\lambda }}_{i}=J^{-\frac{1}{3}}\lambda_{i}$, ${\bar{\lambda }}_{1}{\bar{\lambda }}_{2}{\bar{\lambda }}_{3}=1$, $\mu_{i}$ are the shear moduli parameters, $\alpha_{i}$ and $D_{i}$are material parameters.
Yeoh model^[^ ^217^ ^]^	$W=\sum_{i=1}^{3}c_{i0}{\left(\tilde{I_{1}}-3\right)}^{i}+\sum_{1}^{N}\frac{1}{D_{i}}{\left(J_{el}-1\right)}^{2i}$ where $2c_{10}=\mu .$ $c_{i0}$ and D are temperature‐dependent material parameters.

#### 2.2.4 Viscoelastic Material Modeling

Linear viscoelastic behavior can be described as a combination of an elastic solid and a viscous fluid. The stress–strain relationship in a linear viscoelastic material is in the form of a convolution integral that can be written as

(7) $$\begin{array}{c} \sigma (t)=\int_{0}^{t}G(t-\tau )\frac{d\varepsilon (\tau )}{\text{dτ}}d\tau \end{array}$$

For a simple harmonic shear strain of the form

(8) $$\begin{array}{c} \gamma \left(t\right)=\gamma_{0}\sin\left(\omega t\right) \end{array}$$

where $\gamma_{0}$ is the amplitude of the shear strain, ω is the frequency. Substituting Equation (8) into (7) yields the shear modulus, which can be written as the real and imaginary components of a complex shear modulus of the form.

(9) $$\begin{array}{c} G^{*}\left(\omega \right)=G^{\prime }\left(\omega \right)+iG^{\prime \prime }\left(\omega \right) \end{array}$$

where $G^{\prime }$ is known as the storage modulus and measures the stored energy in the material due to deformation. $G^{\prime \prime }$ is the loss modulus, representing the energy dissipated during deformation.

The phase lag, *ϕ*, called the loss angle, is expressed as

(10) $$\begin{array}{c} \phi =\tan^{-1}\left(\frac{G^{\prime \prime }}{G^{\prime }}\right) \end{array}$$

In the time domain, the shear modulus is often expressed as a generalized Maxwell model using a Prony series as

(11a) $$\begin{array}{c} G\left(t\right)=G_{\infty }+\sum_{n=1}^{p}G_{n}e^{-\frac{t}{\tau_{n}}} \end{array}$$

(11b) $$\begin{array}{c} G_{0}=G_{\infty }+\sum_{n=1}^{p}G_{n} \end{array}$$

where $G_{\infty }$ is the long‐term shear modulus and $G_{0}$ is the instantaneous shear modulus of the material. $G_{n}$ is the individual shear moduli Prony term and $\tau_{n}$ is the individual decay time for the Prony term. The Laplace relates the frequency and time‐dependent shear moduli terms transform of the convolution integral in (7) and can be written as

(12a) $$\begin{array}{c} G^{\prime }\left(\omega \right)=\left(G_{0}-\sum_{n=1}^{m}G_{i}\right)+\sum_{n=1}^{m}\frac{G_{n}\tau_{n}^{2}\omega^{2}}{1+\tau_{n}^{2}\omega^{2}} \end{array}$$

(12b) $$\begin{array}{c} G^{\prime \prime }\left(\omega \right)=\sum_{n=1}^{m}\frac{G_{n}\tau_{n}^{2}\omega^{2}}{1+\tau_{n}^{2}\omega^{2}} \end{array}$$

If the complex modulus is known at a specified frequency, and the number of terms in the Prony series is chosen, the Prony terms and decay times can be determined using an optimization algorithm such as the simplex method or least squares fit.

#### 2.2.5 Fractional Viscoelastic Material Model

For materials with a power law behavior, the relaxation modulus of the viscoelastic material can be described as

(13) $$\begin{array}{c} G\left(t\right)=\kappa t^{-\beta } \end{array}$$

where *κ* and *β* are material constants. The power law model in the frequency domain can be written as

(14) $$\begin{array}{c} G^{*}\left(\omega \right)=G^{\prime \prime }\left(\omega \right)+iG^{\prime }\left(\omega \right)=\kappa {\left(i\omega \right)}^{\beta } \end{array}$$

**Figure 4:**
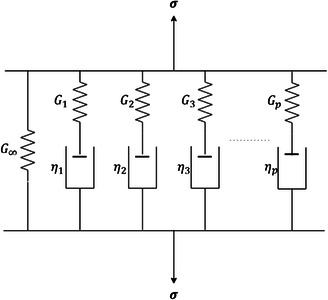
A generalized Maxwell model with ‘p’ series springs and dashpots in parallel. The representation gives rise to a Prony series material model.

The mechanical response of a material with a relaxation modulus described in Equation (14) can be represented by a Scott–Blair linear viscoelastic model commonly known as a springpot.^[^ ^102^ ^]^ The model's physical behavior is intermediate to a spring and a viscous dashpot, see **Figure** **4**. The mathematical implementation of the springpot is obtained using the notion of derivatives of noninteger order or fractional derivatives. The mathematical relationship can thus be written as

(15) $$\begin{array}{c} \sigma (t)=\kappa \frac{d^{\beta }\varepsilon (t)}{dt^{\beta }}\forall (0\leq \beta \leq 1) \end{array}$$

**Figure 5:**
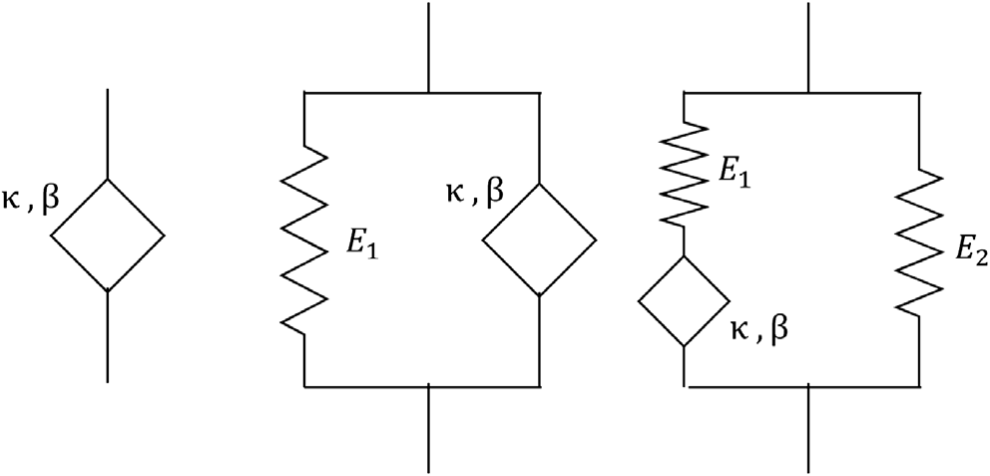
Fractional springpot, Kelvin–Voigt, and fractional linear solid viscoelastic models.

Fractional viscoelastic models can be built by arranging a springpot in series or parallel with a spring, with gives rise to different configurations, as shown in **Figure** **5**, and the stress–strain relationship is shown in **Table** **3**.

**Table 3:** Summary of stress–strain relationships in various viscoelastic material models

Viscoelastic model	Equation of stress to strain
Springpot^[^ ^218^ ^]^	$\sigma (t)=\kappa \frac{d^{\beta }\varepsilon (t)}{dt^{\beta }}\forall (0\leq \beta \leq 1)$
Fractional Kelvin–Voigt^[^ ^219^ ^]^	$\sigma (t)=E_{1}\varepsilon (t)\;+\text{ κ}\frac{d^{\beta }\varepsilon (t)}{dt^{\beta }}\forall (0\leq \beta \leq 1)$
Fractional standard linear solid^[^ ^219^ ^]^	$\begin{array}{c} \sigma (t)+\frac{\kappa }{E_{1}+E_{2}}\frac{d^{\beta }\sigma (t)}{dt^{\beta }}\\ \;=\frac{E_{1}E_{2}}{E_{1}+E_{2}}\varepsilon (t)+\frac{\kappa }{E_{1}+E_{2}}\frac{d^{\beta }\varepsilon (t)}{dt^{\beta }}\forall (0\leq \beta \leq 1) \end{array}$

A generalization of derivatives of noninteger orders can be obtained in a branch of mathematics called fractional calculus using the Caputo derivative.

(16) $$\begin{array}{c} \frac{d^{\beta }\varepsilon (t)}{dt^{\beta }}=\frac{1}{\Gamma (1-\beta )}\int_{0}^{t}{\left(t-\tau \right)}^{-\beta }\frac{d\varepsilon (\tau )}{d\tau }d\tau \end{array}$$

The stress–strain relationship for a springpot can be written as

(17) $$\begin{array}{c} \sigma (t)=\frac{\kappa }{\Gamma (1-\beta )}\int_{0}^{t}{\left(t-\tau \right)}^{-\beta }\frac{d\varepsilon (\tau )}{d\tau }d\tau \end{array}$$

For a 3D finite‐element model, the relaxation matrix can be split into its volumetric and deviatoric components,^[^ ^20^ ^]^

(18) $$\begin{array}{c} G_{ijkm}\left(t\right)=\left(K_{R}\left(t\right)-\frac{2}{3}G_{R}\left(t\right)\right)\delta_{ij}\delta_{km}+G_{R}\left(t\right)\left(\delta_{ik}\delta_{jm}+\delta_{im}\delta_{jk}\right) \end{array}$$

where $\delta_{ij}$ is the Kronecker delta function. $G_{R}\left(t\right)$ and $K_{R}\left(t\right)$ are the deviatoric and volumetric power‐law functions, respectively. The stress–strain relationship in Equation (17) can thus be written for a 3D problem as

(19) $$\begin{array}{c} \sigma_{ij}=\frac{1}{\Gamma (1-\beta )}\int_{0}^{t}\left(K_{\beta }-\frac{2}{3}G_{\beta }\right)\delta_{ij}\dot{\varepsilon_{kk}}(t)t\\ \;+\frac{1}{\Gamma (1-\beta )}\int_{0}^{t}G_{\beta }(\dot{\varepsilon_{ij}}(t)+\dot{\varepsilon_{ji}}(t))dt \end{array}$$

The fractional viscoelastic model can be implemented numerically using the Grunwald–Letnikov operator.^[^ ^101^, ^103^, ^104^, ^105^, ^106^ ^]^ For a 3D state of stress, $\sigma \left(t\right)=\left[\sigma_{11}\;\sigma_{22}\;\sigma_{33}\;\tau_{23}\;\tau_{31}\;\tau_{12}\right]$, the stress in the ij direction at the end of the k+1 interval is given by

(20) $$\sigma_{ij}^{k+1}=\left(K_{\beta }\;-\;\frac{2G_{\beta }}{3}\right){\left(\frac{1}{\Delta t}\right)}^{\beta }\sum_{l=1}^{k+1}\varphi_{l}\varepsilon_{qq}((k+1-l)\Delta t)+2G_{\beta }{\left(\frac{1}{\Delta t}\right)}^{\beta }\sum_{l=1}^{k+1}\varphi_{l}\varepsilon_{ij}((k+1-l)\Delta t)$$

where $k=\frac{\text{Total time}}{\Delta t}$, the number of iterations, total time is the time of simulation, Δt is the time step, $\varepsilon_{\text{kk}}$ is the volumetric strain, and $\phi_{l}$ are the Grunwald coefficients, which can be calculated as^[^ ^22^ ^]^

(21) $$\begin{array}{c} \phi_{l+1}=\frac{\left(k-1-\beta \right)}{k}\phi_{l},\;\phi_{1}=1 \end{array}$$

### 2.3 Hard Composite MSM

#### 2.3.1 Data Acquisition: Experimental Methods

The development of computational materials science relies heavily on sound experimental and data acquisition techniques, which provide real‐world base parameters for meaningful numerical results. Material informatics is a critical area whereby data preparation is done by considering all relevant explanatory variables. After data collection, data preprocessing follows, which involves data formatting, cleaning, and sampling steps, respectively. Feature selection is an essential aspect of the data preparation process, in which only relevant input features are retained while others are deleted to consolidate input parameters for the numerical models.^[^ ^15^ ^]^ Statistically significant constituent data from microscopic experimental analysis of fiber‐reinforced composite specimens need also be acquired. Experimental protocols are required to ensure data consistency and reliability when acquiring fiber/matrix and interraster cohesive composite material properties. Some of the popular approaches for data acquisition that have been put forward in recent times are as follows. Full‐field strain measurement data acquisition utilizing the GOM‐Aramis digital image correlation (DIC) method for longitudinal, transverse, and shear load cases,^[^ ^107^ ^]^ published material databases (such as from national standardization organizations), supplier composites datasheets,^[^ ^108^ ^]^ experimental data obtained using a piezospectroscopic technique,^[^ ^109^ ^]^ etc. State‐of‐the‐art sensors such as fiber Bragg grating (FBG) and strain‐sensing compound basic parallelogram mechanism (CBPM)‐FBG holders have been shown to have superior sensitivity during the MB tests for both synthetic and natural composite filaments.^[^ ^110^ ^]^ Recently, X‐ray computed tomography (X‐ray CT) images of carbon fiber‐reinforced polymer (CFRP) have been used to generate microscale FEM.

X‐ray and micro‐CT is another popular imaging tool to characterize materials and generate 3D visualizations of objects nondestructively. Timelapse imaging methods also help to monitor damage accumulation and cracks. Yu et al.^[^ ^111^ ^]^ discussed an in‐depth number of strategies aimed at increasing the capability of X‐ray CT to detect composite damages. X‐ray‐based CT systems can generate highly accurate 3D inspections of fiber architectures, manufacturing defects, and in‐service damage accumulation nondestructively to avoid the need for primitive mechanical sectioning techniques.^[^ ^112^ ^]^ Ali et al.^[^ ^113^ ^]^ present a comprehensive review of X‐ray computed tomography, which has helped build several 2D and 3D models of liquid composites to detect geometrical and permeability change, fiber orientation variations, and yarn densities when subjected to shear or compaction loads. Digital volume correlation (DVC) has also been proposed to study deformation behavior in epoxy‐based composites; sub‐voxel registration schemes are emerging solutions to attain subvoxel‐level resolutions.^[^ ^114^ ^]^ This technique does not always guarantee an exact match and is validated with FEA results to check correct pairs between DVC volumes.^[^ ^115^ ^]^ As Rashidi et al.^[^ ^116^ ^]^ explain in their in‐depth review, an optimized X‐ray CT (micro‐CT) workflow of composites can assist in generating surface or volume meshes for the MSM of underlying deformation mechanisms, providing qualitative and quantitative compositional information.

Data filtering methods like overlapping stack filtering followed by a local fiber tracking step^[^ ^117^ ^]^ have proven helpful in improving input data quality for numerical simulations. DIC for analyzing deformation mechanisms under transverse compression in a fiber‐reinforced composite^[^ ^118^, ^119^ ^]^ has also gained popularity as one of the data preprocessing techniques. Regulated experimental test data such as load–displacement data deployed for comparison with numerical results^[^ ^120^ ^]^ and grid indentation analysis^[^ ^121^ ^]^ has helped in many inverse and hybrid material model schemes to obtain optimized input parameters for numerical analysis. Advancements in sensor technology, standardization of experimental procedures, quality control, and preventing data leaks/losses would be crucial to attaining high‐fidelity computational models. Automation and removal of latencies in data collection would pave for more extensive, faster, and more accurate experimental results to function as fuel for computational models.

#### 2.3.2 Hard Composites: Material Modeling

The CM to describe a linear elastic solid is the generalized “Hooke's law,” which relates Cauchy stress components with nine deformation components. The stress $\left(\sigma_{ij}\right)$, strain ($\varepsilon_{kl})$, stiffness ($Q_{ijkl})$, and compliance matrices ($S_{ijkl})$ for a material are represented by Equation (22) and (23). The material's constitutive laws govern the physical characteristics of composites, such as conductivity, diffusivity, electric permittivity, magnetic permeability, and electric conductivity. These laws are mathematical idealizations of such behavior, and in this section, some of the predominant models will be outlined. Hard composites commonly can be categorized as anisotropic, transverse isotropic, and orthotropic.

(22) $$\sigma_{i}=Q_{ijkl}\varepsilon_{kl}$$

(23) $$\varepsilon_{kl}=S_{ijkl}\sigma_{ij}$$

Due to the heterogeneous nature of composites, linear elastic behavior of anisotropic composites is very common, where their physical properties are directional. **Table** **4** lists the stress–strain relationship for prominent composite material model types. Furthermore, imposing symmetric requirements can reduce elastic components to 21. Stress–strain ratios can also describe anisotropic materials, as put forward by Azevedo.^[^ ^122^ ^]^ When composites are modeled to be orthotropic, the three symmetry planes help reduce the number of coefficients from 21 to 9 (since angular deformations are independent of normal stress, linear deformation independent of tangential stress, and tangential tension causes only angular deformation in the plane in which it acts). Another way to model composites could be as transverse isotropic material, an orthotropic material with isotropy in one of the planes of symmetry (i.e., the same property in all directions in this plane). The total number of independent coefficients is now reduced from 9 to 5.

**Table 4:** Summary of stress–strain relationships for common constitutive material models

Composite material types/model	Equation of stress to strain	References
Generalized stress–strain relationship (stiffness matrix)	$\left\{\begin{array}{c} \sigma_{11}\\ \sigma_{22}\\ \sigma_{33}\\ \sigma_{23}\\ \sigma_{13}\\ \sigma_{12} \end{array}\right\}=\left[\begin{array}{cccccc} Q_{11} & Q_{12} & Q_{13} & Q_{14} & Q_{15} & Q_{16}\\ Q_{21} & Q_{22} & Q_{23} & Q_{24} & Q_{25} & Q_{26}\\ Q_{31} & Q_{32} & Q_{33} & Q_{34} & Q_{35} & Q_{36}\\ Q_{41} & Q_{42} & Q_{43} & Q_{44} & Q_{45} & Q_{46}\\ Q_{51} & Q_{52} & Q_{53} & Q_{54} & Q_{55} & Q_{56}\\ Q_{61} & Q_{62} & Q_{63} & Q_{64} & Q_{65} & Q_{66} \end{array}\right]\left\{\begin{array}{c} \varepsilon_{11}\\ \varepsilon_{22}\\ \varepsilon_{33}\\ 2\varepsilon_{23}\\ 2_{13}\\ 2\varepsilon_{12} \end{array}\right\}$ 36 independent coefficients, the symmetry between $\sigma_{ij}$ and $\sigma_{ji}$, and $\varepsilon_{kl}$ and $\varepsilon_{l}$	[4, 220]
Generalized Hooke's law for anisotropic materials	$\left\{\begin{array}{c} \varepsilon_{11}\\ \varepsilon_{22}\\ \varepsilon_{33}\\ 2\varepsilon_{23}\\ 2\varepsilon_{13}\\ 2\varepsilon_{12} \end{array}\right\}=\left|\begin{array}{cccccc} \frac{1}{E_{1}} & -\frac{\upsilon_{21}}{E_{2}} & -\frac{\upsilon_{31}}{E_{3}} & \frac{\rho_{2311}}{G_{23}} & \frac{\rho_{1311}}{G_{13}} & \frac{\rho_{1211}}{G_{12}}\\ -\frac{\upsilon_{12}}{E_{1}} & \frac{1}{E_{2}} & -\frac{\upsilon_{32}}{E_{3}} & \frac{\rho_{2322}}{G_{23}} & \frac{\rho_{1322}}{G_{13}} & \frac{\rho_{1222}}{G_{12}}\\ -\frac{\upsilon_{13}}{E_{1}} & -\frac{\upsilon_{23}}{E_{2}} & \frac{1}{E_{3}} & \frac{\rho_{2333}}{G_{23}} & \frac{\rho_{1333}}{G_{13}} & \frac{\rho_{1233}}{G_{12}}\\ \frac{\rho_{1123}}{E_{1}} & \frac{\rho_{2223}}{E_{2}} & \frac{\rho_{3323}}{E_{3}} & \frac{1}{G_{23}} & \frac{\rho_{1323}}{G_{13}} & \frac{\rho_{1223}}{G_{12}}\\ \frac{\rho_{1113}}{E_{1}} & \frac{\rho_{2213}}{E_{2}} & \frac{\rho_{3313}}{E_{3}} & \frac{\rho_{2313}}{G_{23}} & \frac{1}{G_{13}} & \frac{\rho_{1213}}{G_{12}}\\ \frac{\rho_{1112}}{E_{1}} & \frac{\rho_{2212}}{E_{2}} & \frac{\rho_{3312}}{E_{3}} & \frac{\rho_{2312}}{G_{23}} & \frac{\rho_{1312}}{G_{13}} & \frac{1}{G_{12}} \end{array}\right|\left\{\begin{array}{c} \sigma_{11}\\ \sigma_{22}\\ \sigma_{33}\\ \sigma_{23}\\ \sigma_{13}\\ \sigma_{12} \end{array}\right\}$	[122]
Orthotropic material	$\left\{\begin{array}{c} \varepsilon_{11}\\ \varepsilon_{22}\\ \varepsilon_{33}\\ 2\varepsilon_{23}\\ 2\varepsilon_{13}\\ 2\varepsilon_{12} \end{array}\right\}=\left[\begin{array}{cccccc} \frac{1}{E_{1}} & -\frac{\upsilon_{21}}{E_{2}} & -\frac{\upsilon_{31}}{E_{3}} & 0 & 0 & 0\\ -\frac{\upsilon_{12}}{E_{1}} & \frac{1}{E_{2}} & -\frac{\upsilon_{32}}{E_{3}} & 0 & 0 & 0\\ -\frac{\upsilon_{13}}{E_{1}} & -\frac{\upsilon_{23}}{E_{2}} & \frac{1}{E_{3}} & 0 & 0 & 0\\ 0 & 0 & 0 & \frac{1}{G_{23}} & 0 & 0\\ 0 & 0 & 0 & 0 & \frac{1}{G_{13}} & 0\\ 0 & 0 & 0 & 0 & 0 & \frac{1}{G_{12}} \end{array}\right]\left\{\begin{array}{c} \sigma_{11}\\ \sigma_{22}\\ \sigma_{33}\\ \sigma_{23}\\ \sigma_{13}\\ \sigma_{12} \end{array}\right\}$	[122, 220]
Transverse isotropic material	$\left\{\begin{array}{c} \varepsilon_{11}\\ \varepsilon_{22}\\ \varepsilon_{33}\\ 2\varepsilon_{23}\\ 2\varepsilon_{13}\\ 2\varepsilon_{12} \end{array}\right\}=\left[\begin{array}{cccccc} \frac{1}{E_{1}} & -\frac{\upsilon_{21}}{E_{2}} & -\frac{\upsilon_{31}}{E_{1}} & 0 & 0 & 0\\ -\frac{\upsilon_{12}}{E_{1}} & \frac{1}{E_{2}} & -\frac{\upsilon_{12}}{E_{1}} & 0 & 0 & 0\\ -\frac{E_{13}}{E_{1}} & -\frac{\upsilon_{21}}{E_{2}} & \frac{1}{E_{1}} & 0 & 0 & 0\\ 0 & 0 & 0 & \frac{1}{E_{12}} & 0 & 0\\ 0 & 0 & 0 & 0 & \frac{1}{G_{13}} & 0\\ 0 & 0 & 0 & 0 & 0 & \frac{1}{G_{12}} \end{array}\right]\left\{\begin{array}{c} \sigma_{11}\\ \sigma_{22}\\ \sigma_{33}\\ \sigma_{23}\\ \sigma_{13}\\ \sigma_{12} \end{array}\right\}$	[220, 221, 222]

In Table 4, in the expression for mathematical representation of Hooke's law for anisotropic materials, symbol ***E*** is Young's modulus, ***G*** is the shear modulus, ***υ*** is the Poisson's coefficient, and ***ρ*** is the angular/linear deformation.

#### 2.3.3 Failure Constitutive Model for Composites

Failure in composites depicts a scenario where there could be a global or local failure. Damage and failure in polymer and FRP or laminate‐based composites have gained much attention from the computational community. Some of the prominent failure models are the maximum normal stress (Rankine), maximum shear stress (Tresca), maximum normal strain Saint–Venant), and maximum strain theory (Von Mises). Several failure criteria are available in the literature to describe failure in composites: Hill, Tsai‐Hill, Tsai‐Wu, Hashin‐Rotem, Hashin, maximum stress, Hoffmann, maximum strain, Hou, Puck–Schürmann, Chang‐Chang, Linde, LaRC03, LaRC04, Maimí, Hart‐Smith, and Yeh‐Stratton to name a few.^[^ ^4^ ^]^

Guo et al.^[^ ^123^ ^]^ in their 2021 review on CMs for FEA in composites, discuss that for structural performance‐based analyses of composites, the CM are divided into four major groups such as: 1) formulations based on CM for homogeneous materials (isotropic and orthotropic) models of damage, plasticity, viscoplasticity, and cohesive fracture, 2) formulations based on the composition of isotropic or orthotropic phenomenological models, 3) formulation based on homogenization of single‐/multiple‐scale isotropic or orthotropic phenomenological models, and 4) kinematic formulations for structural elements using several isotropic or orthotropic phenomenological models.” They also enlist some popular damage formulations based on CMs, such as the CDM, cohesive damage model, mixture theory model, MS model, and nonorthogonal CM.

In Table 4, for CDM expression, ***E***_**1**_ ***, E***_**2**,_ and ***v***_**12**_ denote the in‐plane elastic orthotropic properties, wherein subscript 1 denotes the longitudinal (*fiber*) direction, and 2 denotes the transverse (*matrix*) direction; ***d***_**1**_ is associated with longitudinal (fiber) failure; ***d***_**2**_ damage variable associated with transverse matrix cracking; ***d***_**6**_ is the damage variable associated with longitudinal and transverse cracks; ***α***_**11**_ and ***α***_**22**_ are the coefficients of thermal expansion in the longitudinal and transverse directions, respectively; ***β***_**11**_ and ***β***_**22**_ are the coefficients of hygroscopic expansion in the longitudinal and transverse directions, respectively; and **Δ** ***T*** and **Δ** ***M*** are the differences in temperature and moisture content concerning the corresponding reference values. CDM is widely used to model damage and predict the corresponding isotropic/anisotropic stiffness degradation and damage evolution. CDM investigates the macromechanical effects caused by generating and developing microdefects inside materials. The cohesive damage zone model combines tractions and displacement jumps at interfaces where cracks occur. The advantages of cohesive zone models are their simplicity and uniformity of crack initiation and propagation in composite structures.

Mixture theories are deployed to simulate the non‐linear anisotropic behavior of composites at high strains. Truesdell et al.^[^ ^124^ ^]^ first studied the mixture theory used in many further studies. In **Table** **5**, for the mixture theory stress–state equation, notations are ***m*** denotes the density of the composite, ***m***_***c***_ is the density of each of the phases (first proposed by Car et al.^[^ ^125^ ^]^), ***ψ***_***c***_ is the free energy corresponding to each constituent phases, ***k***_***c***_ is the volumetric participation coefficient. Finally, the nonorthogonal constitutive group of models considers the composites’ structural elements, emphasizing the microstructure to build the model. This model is especially useful for studying woven fabric damage mechanisms that undergo small surface extensions and large angular variations. For the nonorthogonal model described in Table 5, $\left[S^{*}\right]$ is the matrix containing component stress tensor, compliance constants (*S*_*ij*_ *, S*_*ijk*_ *, S*_*ijkl*_), the second‐order constants (*S*_11_ *, S*_22_ *, S*_12_ *, S*_66_), and others, including the fiber orientation angle *θ*, measured with respect to the x‐axis after applying necessary transformations to the nonlinear Eulerian stress–strain coordinates. The small deformation nonorthogonal model was proposed by Yu et al.^[^ ^126^ ^]^ in their proposed equation (Table 5), $E^{\alpha }=\;E^{\alpha }A^{\alpha }$, $A^{\alpha }$ and $E^{\alpha }$ are cross‐sectional area and elastic modulus of yarn in α direction, c is the thickness of composite. Further details on their model can be found in ref. [123].

**Table 5:** Summary of stress–strain & free energy relationships for damage in composites

Damage model for composites	Equations to describe the free‐energy density function/stress state relationships	References
CDM	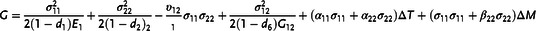 G depicts the complementary free energy density	[223, 224]
Cohesive damage model	$\psi \left(\Delta ,d\right)=\left(1-d\right)\psi^{0}\left(\Delta_{i}\right)-d\psi^{0}\left({\bar{\delta }}_{3i}-\Delta_{3}\right)$ Here, ⟨x⟩ is the McCauley operator; ***d*** is a scalar damage variable; and ***ψ***_**0**_ is the free energy per unit surface area.	[225, 226]
Mixture theory model	Stress state: $\sigma =m\partial \psi \partial e=\Sigma_{c=1}k_{c}m_{c}\partial \psi_{c}\partial e=\Sigma_{c=1}k_{c}m_{c}\sigma_{c}$	[123, 124, 125]
Nonorthogonal constitutive model	Stress–strain relation for flexible materials: $\left\{e\right\}=\left[S^{*}\right]\left\{\sigma \right\}$ For small deformation, taking kinematics and fiber properties into account, modified stress–strain relationship for fabric‐reinforced thermoplastic composites $\sigma_{xx}\sigma_{yy}\sigma_{xy}=E^{\alpha }bc+\Gamma a\bar{h}a^{2}c\Gamma a\bar{h}b^{2}c\Gamma a\bar{h}abc\Gamma b\bar{a}a^{2}c\Gamma b\bar{a}b^{2}c+E^{\beta }\bar{a}c\text{Γb}\bar{a}abc\Gamma b\bar{h}a^{2}c\Gamma b\bar{h}b^{2}cc\Gamma b\bar{h}abc\varepsilon_{xx}\varepsilon_{yy}2\varepsilon_{xy}$ where $\Gamma ={\text{ A}}^{\gamma }E^{\gamma }/{\left(a^{2}+b^{2}\right)}^{3/2},$ $a=\bar{b}\cos\phi$, $b=\bar{b}\sin\phi$	[126, 227, 228]

The models in Table 5 are not an exhaustive list of damage models for composites. For the scope of this article, only a few selected CMs and popular failure/damage models have been included to introduce the mathematics behind the modeling of complex composite mechanics in numerical solvers. These codes use custom subroutines or self‐defined material codes. Many of these formulations are deployed under the hood in solvers, as we will learn from popular composite MS models in the following sections.

#### 2.3.4 Model Setup: Key Inputs, Boundary Conditions, Outputs

##### Modeling Objectives

Most MS computational models are developed to define composite material behavior based on their constituent phases and mechanical properties. These objectives are frequently realized using materials’ mechanisms and elasticity theories based on repeated repeating unit cells (RUC) or RVEs. From a modeling standpoint, a perfect bond between the fiber and matrix constituent phases is assumed, which may not be ubiquitously true. Another common objective of computational models is to validate experimental results in the field and match predicted values. These broad goals are achieved by various computational frameworks ranging from atomistic scale to macro‐/part scales. MS models simulate these composite behavior across multiple time and length scales.^[^ ^127^, ^128^ ^]^

##### Popular Tools/software Packages

Increased computational power and data processing capabilities have been instrumental in developing modern‐day complex, accurate, and efficient MS numerical models. Some of these standard tools and software packages are Gaussian (focused on quantum chemical calculations), Materials Studio (for quantum, classical, and mesoscale computations), and ABAQUS/Ansys/Altair/COMSOL/Star CCM + NX solver (for FEM, CFD, and multiphysics calculations), and Codes such as Cambridge Serial Total Energy Package (CASTEP) and Vienna Ab initio Simulation Package (VASP) have become available from specialized academic groups to industrial users and experts. MSM protocols, such as the OCTA project and the VOTCA (Versatile Object‐oriented Toolkit for Coarse‐graining Applications) toolkits, have also propelled computational materials research. Please note that this list is not exhaustive, and many more tools/packages/codes are being introduced. A few additional codes and their significant contributions have also been summarized in Section 2.3.4. (**Table** **6** and **7**).

**Table 6:** Summary of the critical model contributions in hard‐tissue composite materials modeling spanning across micro‐, meso‐, and macroscale. Some additional state‐of‐the‐art interscale models are outlined. Key model variables (inputs, B.C./setup, and output) are referenced

Scale	Model name/Examples	Input	Setup	Output	References
Microscale	Fibrous modeling (RUC/RVE)	Fiber/matrix/interface VF Spatial distribution	RUC and its constituents	Composite unit cell behavior (Homogenization)	[4, 229]
	Two‐scale asymptotic homogenization theory	Unit cell in fabric weaves of composites, fiber VFs	Boundary value problems (BVP), 3D FEM for determining thermal properties	Orthotropic mechanical stiffness tensor, thermal expansion, thermal conductivity	[230]
	ASC technique	3D RVE, FAR), fiber volume fraction (FVF)	3D periodic boundary conditions (PBC)	Analyze composites with random fibers (elastic properties)	[231, 232]
	Micromechanical FEM	Delaunay triangulation method, failure criteria defined, compression load	Periodic boundary conditions, local material properties, fiber orientation, mixed loading	Microdamage evolution in the z‐composite, in‐plane compressive strengths	[233, 234, 235, 236, 237, 238]
	Microgeometrical modeling	FARs and VFs, fiber distribution, homogeneity, laminated random strand method	3D micro‐FEM, Windowing‐type analysis	Elastic properties of in‐plane random FC (stiffness tensor)	[239]
	Digital element approach (DEA), truss element‐based approach, and beam element‐based approach	Elastic element, beam stiffness. beam elements modeled as elastic–plastic material model	3D‐woven fabric geometry (virtual fibers). relative static sliding contacts	Fabric deformation and microstructure modeling predict fabric properties	[132, 240, 241]
	Composite Damage and failure modeling	Failure or damage criteria	Inverse order modeling	Fiber rupture, matrix failure, and interface detachment	[10, 130]
	Compressive failure – influence of voids and imperfections	Cohesive zone model, displacement load applied.	Reduced mechanical model composite (focusing on one unique void)	Alignment of fibers, as well as fiber–matrix bonding, influence failure initiation	[242, 243]
	Micro‐mechanical FEM	Volume average method for fused deposition modeling (FDM)	Morphology, raster orientations	Layer height influence on FDM parts’ elastic properties	[244, 245]
		RVE or RUC, isotropic laminate material model	Mixed loading conditions, quasistatic	Elastic deformation, stiffness, stress prediction, compression strength, debonding, frictional sliding, rupture, and sliding of pins in z‐directional composites	[236, 246, 247, 248, 249, 250]
	Fracture model using FEM for UD‐CFRP	Frictional heating, plastic energy dissipation, fiber orientation, square packing	Damage‐based fracture method; thermomechanical coupling model	Deformation mechanism and cutting force, surface roughness	[251]
Mesoscale	Composite damage model, Bottom‐up	Interlaminar properties Unit cell setup Laminate layup	Homogenized microscale properties	Homogenized laminate behavior, Failure envelope	[252, 253]
	MC (MC simulation) and FEM	VF, Homogenization (random aggregate structure), Unit cells (UC)	Isotropic damage model, FEM (FEAP code), P.B.C.,	Effective properties of concrete composites. progressive degradation	[254, 255]
	Weaving simulation models, digital chain method: surrogate modeling (Kevlar, carbon–Kevlar hybrid woven fabric)	Yarn path, cross section, linear elastic model–fiber VF, friction coefficient	WiseTex, TexGen codes, yarn motion, and cross‐section information, Abaqus, Explicit solver	Weaving process, predict yarn geometries, topology, stiffness, resistance to fracture, damage, or fatigue	[133, 256, 257, 258, 259, 260, 261]
	Mapping and reverse mapping	Radial distribution function, end‐to‐end distance, matching densities	Adaptive umbrella sampling, thermodynamic reweighting	Calibration of polymer chain into particular systems	[59, 262]
	Wang–Landau method	Histogram of sample states distribution reweighting	Random‐walk algorithm	Full DOS, heat capacity, Surface‐induced crystallization, multilayer coil‐globule transition	[263, 264, 265]
	DPD	Density beads, Hydrodynamic behavior parameters	ESPResSo Code, Materials studio	The flow of sphere agglomerate particles	[266, 267, 268, 269, 270]
		Uniaxially oriented fibers, coarse‐grained CNTs, shear forces in fibers (varying aspect ratios)	MC method	Electrical impedance, modeling of mixed systems carbon nanotubes, percolation thresholds	[271, 272, 273]
	DDFT	External potential variation	MesoDyn package‐coupled Langevin equations	Particle density distributions, free energy minimized	[274, 275]
	PFM	Free‐energy functional, Poisson seeding algorithm	Nucleation rates, interparticle spacing, particle size, microstructure	Solidification, nucleation, dislocation dynamics	[173, 276]
	Random walk model	Natural yarn & fibers RVE geometries (FibrilPack)	FORTRAN code, TPI, Axial strain, creating natural yarn fibers	Final yield strength and modulus of the yarn, stress localization	[148]
	Fiber Walk	Twist, fiber diameter, yarn diameter, and number of fibers, mechanical properties and microstructural geometry	Dash general contact algorithm, Nonconstant displacement rate B.C. quasistatic pull	Fracture strength (tenacity), stress–strain distribution	[147, 277]
	Analytical model + FEM	Twist angle, yarn diameter, filament geometry, and filament mechanical properties	TPI, RVE model	Yarn tenacity (strength/weight ratio) as a function of twist ratio	[146, 148]
	XCT analysis + Voxel 3D model. Model types: realistic voxel type, stochastic type, and simplified model	Micro‐CT images, 3D images, 3D voxel models (using FEM or FVM)	Shear loads, compaction studies, edge detection image processing	Fiber orientation changes, yarn waviness, permeability, and geometrical changes in 3D fibers	[113, 278, 279, 280]
	Elastic CM (linear elastic mechanical properties)	Filament properties, yarn geometry, twist angle, yarn closed packed radius, and relaxation due to twist force	Ends of yarn fixed, twist applied, constant force	Stress and strain gradient. Failure strains as a function of TPI and radius	[146]
Macroscale	CDM	Laminate properties, component geometry, component connection	Composite grid method (HFAC)	Structural behavior/continuum shell properties	[281, 282, 283]
	Finite‐element models	Isotropic material properties, Square RVE	Generalized plain strain method	Stress analysis, heat, or mass transfer	[284]
		Stress components, orthotropic material properties	Tsai‐Hill, EHM material model, quasistatic process	Laminate failure analysis, shear stress failures, cutting, and thrust force for UD‐FRP composites	[285]
	Quasicontinuum method	The energy of each element in FEM	Remeshing, zero temperature, static equilibrium	Interface structures and deformations	[286, 287, 288]
	Damage modeling in woven composites using FEM	3D Hex cohesive elements, axial stiffness, nonlinear 1D spring elements.	Full FEM, equivalent binary model of the unit cell, scale–coupling framework	Damage in woven composite macrostructures, stiffness degradation, progressive composite damage	[289]
	FFT homogenization	Regular voxel grid (3D images like tomographies)	Lippmann–Schwinger type, macroscopic Newton algorithm	Compute mechanical macroscopic response: localization and softening of material response	[290]
		Spatial distribution of phases (binary or multiphase image), composite voxels	Homogenization problem solved in frequency domain (Neumann algorithm, Lippmann–Schwinger equation: periodic B.C.)	Effective elastic properties: Young's modulus and Poisson's ratio	[291, 292]
		Trilinear hexahedral elements on Cartesian grids	Lippmann–Schwinger equation (tensile load case tests)	Macroscopic elastoplastic response	[293]
		Images of the microstructure	Lippmann–Schwinger's equation, solved iteratively by the Green operator	Overall composite properties, local distribution of stresses and strains	[294]
		Macroscopic strains (periodic boundary condition)	Hashin and Shtrikman (energy principle)	Model periodic elasticity mechanics: variational framework	[295]
		Nonlinear materials (viscoelastic)	“Second‐order” nonlinear homogenization method: pure shear and simple shear load, conjugate/fast gradient methods,‐ numerical homogenization	(crossover‐type Macroscopic behavior): linear and nonlinear properties	[296, 297, 298]
		Volume element (high‐contrast composites)	Augmented Lagrangian method (linear elastic problem)	Investigating effect properties of linear elastic material with voids, two‐phase composites, and voided rigid–plastic materials.	[299]
		Regular voxel grids (from 3D image tomographies)	Arbitrary microstructure and loading (multiaxial load governed by Kuhn–Tucker relations)	Localization and softening, composite failure and progressive damage	[300]
	Direct numerical simulations (DNS)	Deterministic governing equations, material properties (homogenized)	Parallel FEM code, Sierra Multiphysics, FETI‐DP codes, Lagrange multiplier	Macroscale response of polycrystalline polymers, Predicting fatigue and fracture initiation in composites	[301, 302]
Other MS models	Cohesive zone model (CZM)	Traction separation, softening law	Cohesive elements in FEM to simulate crack initiation and propagation	Crack growth, fiber–matrix debonding, and interlaminar delamination, fracture energy of the material	[237, 277, 303, 304]
	RTM6 CM	Frictional cohesive elements, DIC	2D square RVE, Periodic B.C. (PBC)	Strain localization affects	[305]
	FEA–braided composites–thermal analysis	interior braiding angle, pitch length, side length, fiber VF, and yarn VF	RVE (micro‐ and mesoscale), full‐scale braided model	Thermal conductivities of braiding yarns	[306]
Hierarchical modeling	MS (micro–macro) elastic to the plastic damage model	Rate‐independent l tension and compression, shear, combined shear + tension/compression	Homogenization for property transfer (UMAT, VUMAT, in Abaqus)	Impact response of 3D composites	[307]
	Differential quadrature hierarchical finite element method (DQHFEM)	NURBS elements, isotropic material properties (homogenous material)	molecular dynamic simulations and Numerical analysis	Analyze the interface of composite structures	[308]
Hybrid models	FE/MD/TB (tight binding approximation)		Uniaxial tension, brittle fracture	Large‐scale simulation of impact damage	[309]
Large‐scale fracture mechanics models	Accelerated dynamics technique	Pair potential, long‐timescale relaxations, 3D‐woven composite geometry	Collision cascade simulations, chemical rate theory	Radiation damage profile evolution, microcracking	[310, 311]
MS aging and degradation models	Disturbance and the collision algorithm	Temperature, moisture absorption, 3D strains	Genetic algorithms: random fibers, matric, and fiber	Hygrothermal aging behaviors, macroproperty degradation, microstructure evolution	[312]

**Table 7:** Summary of molecular dynamics (MD) models

Input	Tool	Output	Author
Molecular model of MT in axon	LAMMPS with interatomic potential adopted CHARMM36 with CMAP	Damage and failure strains under extremely high strain rates during cavitation bubble implosion	Wu and Adnan^[^ ^313^ ^]^
Coarse‐grained axolemma (lipid bilayer)	GROMMACS with force fields generated using Martini force field for CGMD	Mechanoporation due to applied strains	Montanino et al.^[^ ^314^ ^]^
Fitting atomic model of αβ tubulin‐built MT into cryo‐EM map of a complete MT.	NAMD with CHARMM27 parameters with CMAP corrections	Nonlinear axial stress–strain behavior	Wells and Aksimentiev^[^ ^315^ ^]^
An atomistic‐continuum model via a higher‐order Cauchy–Born rule of MT	Numerical computation for simulation of dynamic response and spectral analysis for long MTs	Vibration characteristics of curved MTs	Xiang and Liew^[^ ^316^ ^]^
A periodic model of actin–spectrin cytoskeleton	LAMMPS with OPLS force field	Atomic stresses at high strain rates.	Kan et al.^[^ ^317^ ^]^
Molecular model of axon membrane‐bound ion channel	GROMACS with all‐atom CHARMM36 force field	Shape parameters of the protein structure	Lau et al.^[^ ^318^ ^]^
An atomistic‐based continuum viscoelastic CM of αβ tubulin	DL‐POLY using AMBER force field	Material properties of MT	Adnan et al.^[^ ^319^ ^]^
CGMD model of the membrane structure of an unmyelinated axon	Numerical computation using CGMD	Material properties of the axon	Zhang et al.^[^ ^95^ ^]^

##### Modeling Approaches

Micromechanical modeling approaches such as the RUC setup model individual fibers and matrix–fiber interfaces to characterize material behavior locally and study defects/interphrasal mechanics. Localized defects include fiber–matrix detachments, complex grain‐boundary deformations (in fiber‐reinforced composites), delamination, and constituent damage mechanics. It, however, comes at a high computational cost due to the very fine mesh necessary to model such complex physics.^[^ ^129^ ^]^ As a result, homogenization aspects in composite modeling are often leveraged. Equivalent homogenous material (EHM) comes under this bracket of modeling approach widely deployed to reduce simulation time. The downside is that they cannot be used to predict localized effects (e.g., fiber–matrix interface damage^[^ ^4^ ^]^). Moving up, the mesoscale approaches account for individual fibrous composite layers geometries, and finally, the macroscale/part‐scale composite laminate effects are considered homogenously.^[^ ^130^ ^]^ A third approach could be a combination of the above two methods to identify the region of interest and the scope of homogenization to develop cost and time‐efficient numerical simulations. Specifically for FEM computations, choosing the appropriate element size and definition is critical to efficiently analyzing complex composite structures. The choice of element type and order depends on the model accuracy requirements and the meshed geometrical features. For incompressible materials, hybrid elements are necessary to capture nonlinear deformations. Defining preprocessing parameters in MS and multiphysics composite systems should be done based on current computational resources and fidelity requirements.

The models summarized in Section 2.3.4. (Table 6 and 7) are not exhaustive to cover the breadth and depth of MSM for hard composites,^[^ ^131^ ^]^ but the intent is to provide a concise overview of techniques across multiple scales. This article complements recent reviews on composite modeling by including a greater breadth of numerical models rather than focusing solely on woven fabrics,^[^ ^132^, ^133^ ^]^ laminated composites,^[^ ^41^ ^]^ or a specific approach^[^ ^18^, ^115^, ^116^, ^134^ ^]^ to composite modeling. As outlined previously, presenting soft^[^ ^135^ ^]^ and hard composite models in the same study would also encourage model transferability and innovation in modeling new emerging composites and heterogenous metamaterials that could have engineered properties from different families of composites.

Homogenization techniques have also emerged in the analysis of composite materials to determine effective material properties at a macroscopic level based on the properties of the constituent phases at a microscopic level. As briefly touched upon in Table 6, fast‐Fourier transform (FFT)‐based homogenization techniques are numerical methods that use FFT algorithms to efficiently compute the homogenized properties of composites. For FFT analyses, composite microstructure representation is done using spatial distribution of phases (binary or multi‐phase image). These spatial distributions are then transformed into frequency domain using FFT. The homogenization problem is then solved in frequency domain where the transformed microstructure is used to compute composite response. Finally, the response plots are transformed back to spatial domain using inverse FFT. Recently, Magri et al.^[^ ^136^ ^]^ proposed a series of numerical simulations on FFT‐based techniques for 3D composites microstructures to develop computational homogenization framework for understanding particle size effect in ductile composites by incorporating low–to‐higher‐order strain gradient plasticity model and damage models. Interested readers of this technique are also encouraged to check out a recent article by Schneider,^[^ ^137^ ^]^ which reviews the current state‐of‐the‐art nonlinear computational homogenization methods using FFT in great detail. Lucarini et al.^[^ ^138^ ^]^ provided a comprehensive review of FFT approaches for micromechanical simulations, covering the basic mathematical aspects and descriptions of approaches including the basic scheme, polarization‐based methods, Krylov approaches, Fourier–Galerkin, and displacement‐based methods. Moreover, their review article also captures several FFT applications which combine existing synergies with experiments and offer insights on extension of FFT models toward dislocation dynamics, multiphysics, and MS composite problem and could be a useful reference for interested readers.

**Figure 6:**
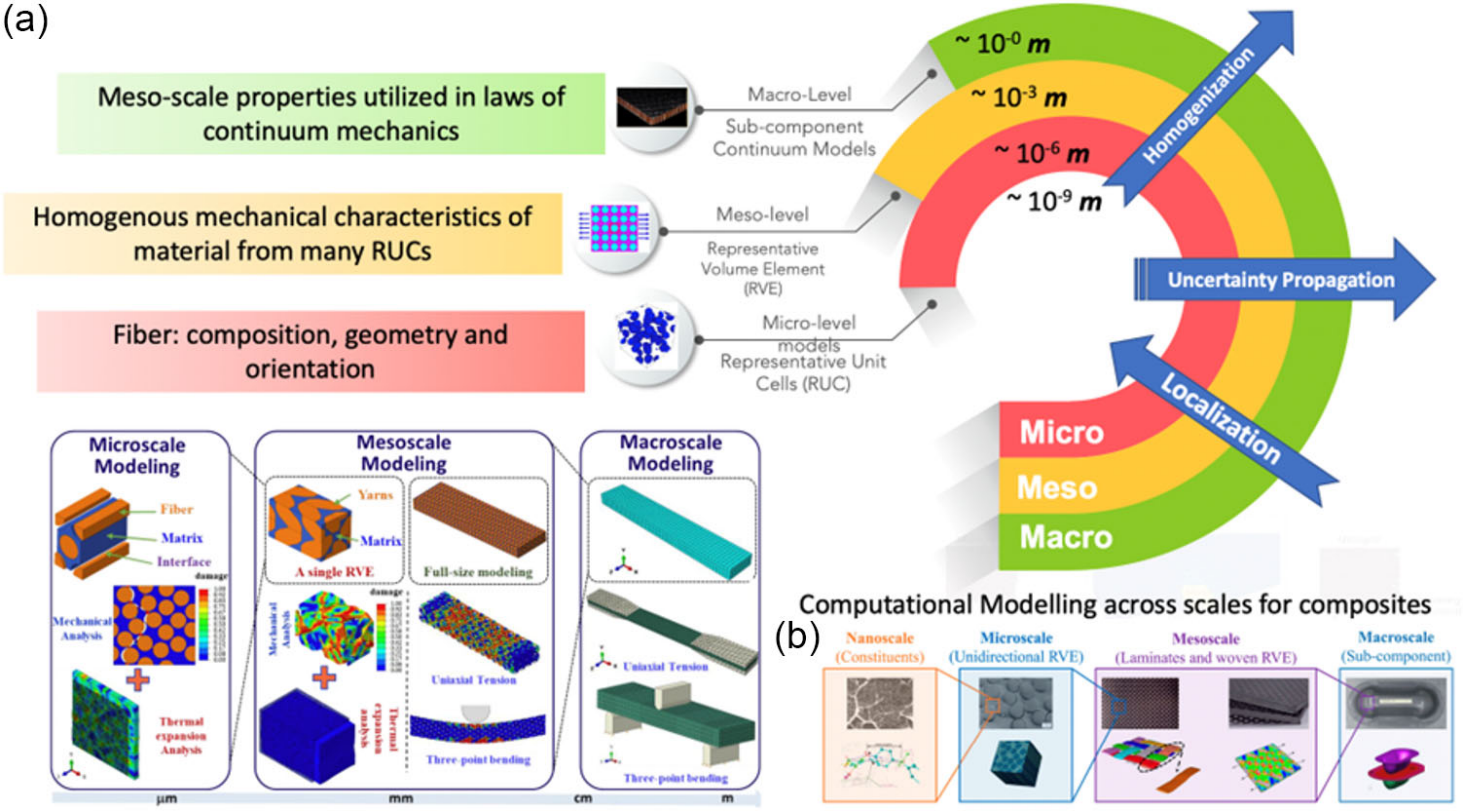
Schematic diagram showing length and timescale ranges of the micro‐, meso‐, and macroscale models. Homogenization and localization of composite material properties from micro‐ to macro‐scale. a) Adaptation of a figure depicting the framework of the MS analysis for 3D braided composites by He et al.^[^ ^211^ ^]^ Copyright 2020, Elsevier. b) Adaptation of a figure from Muzel et al. which describes a schematic view of a four‐scale woven fiber composite with polymer matrix as an example, depicting the computational modeling across scales in composites.^[^ ^4^ ^]^ Copyright 2020, MDPI.

Besides some of the few listed models in Table 7, an in‐depth review from Bargmann et al.^[^ ^139^ ^]^ also outlines seminal work in RVE generation for heterogeneous materials. Their paper discusses physics‐based microstructure generation via kinetic MC methods that link MD simulations to mesoscale models. Emphasis is placed on physics‐based and geometrical models^[^ ^140^ ^]^ for physical processes morphology and on evaluating kinetics across scales that influence microstructure formation and evolution^[^ ^139^ ^]^ in alloys and composites. In their review article, Müzel et al.^[^ ^4^ ^]^ briefly summarized the hierarchical mapping in MS composite models for fiber‐reinforced random forest (RF) composites (**Figure** **6**). In their study, interactions between “Fiber Modules,” “Lamination Modules,” and “FEA” modules spanning across micro‐ to macroscale have been aptly outlined for both UD and bidirectional FR composites. The fiber module is used for setting up the composite material geometrical and material definitions, which are then relayed to the Lamination module for computation of laminated composite‐level properties. Finally, these results are used in the FEA of macroscopic structures to determine part‐level stiffnesses. This encompasses a typical forward or up‐scale MSM workflow.

On the other hand, for inverse model workflows, stress and deformation derived from macroscale models are decomposed at the microscale to determine fiber and matrix constituent‐level material properties. It completes the stress analysis loop. RVE geometry should statistically represent heterogeneity in composite materials. Effective properties derived from RVE analysis denote part‐scale material properties per micro–meso–macro MS separation analysis architecture. For mimicking the randomized dispersion of fibers in composites, the random sequential adsorption algorithm and the MC procedure are recommended for generating artificial RVEs for MS simulations. Similarly, the Automatic searching and coupling (ASC) technique enables 3D RVE generation for composite analysis with random fibers having a broad range of fiber aspect ratios (FARs).

##### Imperfections in Composites

Microvoids and imperfections are common defects generated during the manufacturing and lifecycle of composites. Based on surrounding conditions, microvoids could be matrix voids and interfiber voids. These voids can have detrimental consequences on the load‐bearing capacity of composites. A wide variety of models have been proposed to model imperfections in composites. Sharma et al.^[^ ^141^, ^142^ ^]^ proposed an image‐based FEM. X‐ray tomography was used to explore cracks, voids, and fiber bundle distortion and to reconstruct 3D FE meshes incorporating these defects. These models were studied to analyze the influence of imperfections on homogenized effective moduli of 3D and 4D in‐plane carbon/carbon composites (C/C). Imperfection‐based FEM has also been developed to study yarn waviness, misalignment or nesting of fibers,^[^ ^143^ ^]^ and kinking failures in UD composites. Micro‐CT and MS DIC have also shown promise in characterizing the 3D size, shape, and distribution of voids in fiber‐reinforced composites.^[^ ^144^, ^145^ ^]^ These examples signify the importance of coupled/hybrid modeling approaches as future avenues of imperfection analysis.

#### 2.3.5 Model Assumptions and Limitations

In many RVE geometries for meso‐ and macroscale setups, the fibers are modeled as uniformly distributed and assumed to have the same average diameter. RVEs with higher VFs and large fiber proportions will be generated for modeling composites with spatially distributed random discontinuous fibers. It is challenging to generate RVEs composed of randomly chopped fibers by following manual creation methods because of the 3D spline trajectories, high packing density, tortuosity, and interweaving. Researchers have developed algorithms to model such high‐packing density arbitrary shapes.^[^ ^146^ ^]^ These algorithms randomly orient particles inside a volume and often end up with particle overlapping and limited aspect ratios (length/diameter). The tortuosity of fibers is overlooked, and fibers are assumed to be oriented parallel to minimize initial RVE geometry complexities. For yarn‐composites models, fiber diameter and yarn diameter values are assumed to follow a specific distribution, and average dimensional values are considered in proof‐of‐concept models.^[^ ^147^ ^]^ Many composite materials undergo harsh environmental conditions and modeling such nonlinear multiphysical behaviors still requires much refinement and adjustments to replicate real‐world scenarios. Currently, many numerical models are limited to including only mechanical properties and microstructural geometry. Exhaustive models incorporating multiphysical properties and offering high scalability need still further exploration. When computational models are developed with errors in experimental data when data fitting or statistical models (least square method) are applied, the average, homogenized material model input parameters will contribute to uncertainty propagation in MS simulations (see Figure 6). Values, including statistical deviation in filament stiffness and strength, improve prediction capabilities.^[^ ^148^ ^]^ Incorporating contributions from filament elongations, Poisson's ratio, filament modulus, and appropriate constitutive mathematical material model, especially for multiaxial loaded boundary value composite analyses, is necessary to accurately predict failure strains in yarn or other fiber‐reinforced composites by.^[^ ^149^ ^]^

#### 2.3.6 Gap Assessment

Improving the reliability and repeatability of computational models will be the prime movers in ensuring greater acknowledgment of numerical models in mainstream product development. While computational models are not meant to obviate experimental analysis, high‐fidelity computational models would improve material development workflow faster and with lower costs.

On that notion, a random intertwined fiber geometry in the RVE model via a random walk or other popular algorithms would help closely depict fiber distribution in the matrix. This extends to soft composite meso‐ and macroscale models in which axonal pathways can be better mapped in areas such as the spinal cord and the brain using MRI techniques.^[^ ^148^ ^]^ State‐of‐the‐art data acquisition tools could help minimize errors during experiments. As discussed in Section 1, damage mechanics of composites has gathered much attention from industry and academia. One example is plastic filament yarns (Kevlar), which have been explored^[^ ^146^, ^147^, ^148^, ^149^, ^150^ ^]^ for ballistic and wear‐resistant applications. These materials were analyzed for tenacity, strength, and damage evolution. The study posits that incorporating parameters like filament breakage variance as one of the likely failure points during analysis could improve fit with experimental results and reliability of the micromechanical FEM model. Nonlinear load response analysis also merits further research in developing computational yarn tenacity models.^[^ ^146^ ^]^ In soft materials computational research, significant efforts have been made to characterize BWM response. Within the soft composite research, Wu et al.^[^ ^68^ ^]^ leveraged a ML‐based workflow alongside FEM to generate a hybrid setup to predict material properties using bionic axonal fiber geometry data as voxel input data. This method is far more computationally efficient when compared to solving continuum‐scale FEM models in Abaqus. Hybrid modeling, ROMs, and data science‐driven predictive modeling and sensitivity analyses can also be leveraged for research on yarn, FRP, CRFP, nanocomposites, and other polymer composite materials.

#### 2.3.7 Applications

**Figure 7:**
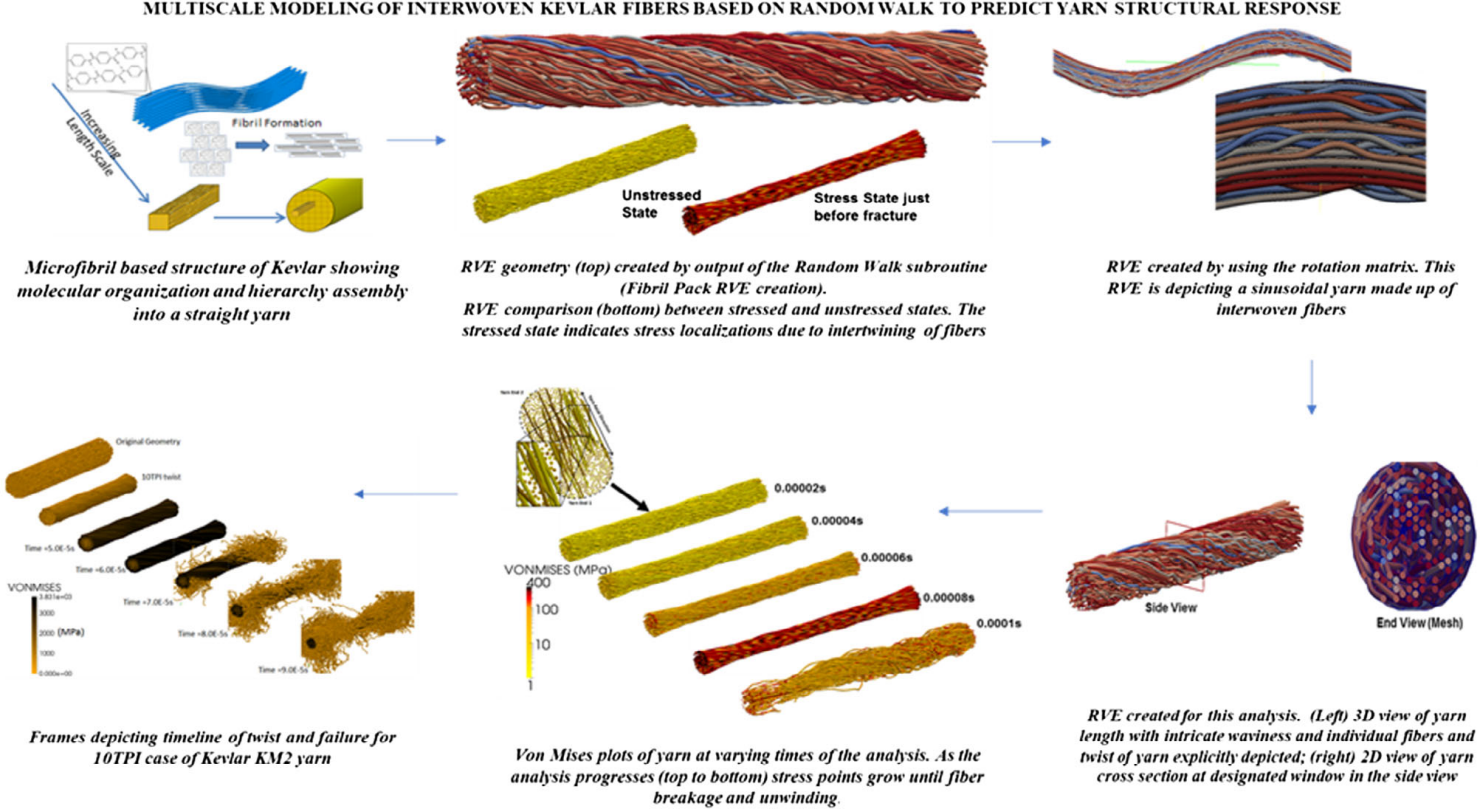
A yarn analysis overview diagram presenting Kevlar fibers‐based numerical modeling research conducted by Stephen Recchia, outlining the RVE geometry creation process (random walk algorithm) and interwoven fibers. Von Mises stress plots for the yarn at varying times and eventual fiber breakage and unwinding. Composite models were developed to depict the timeline for damage and twist failures in Kevlar yarn.^[^ ^146^, ^149^ ^]^

Hard composite fiber mixtures and structures‐based MS models appeal to advanced ballistic and armament structures and biological systems development. Researchers have surmised that using explicit solvers instead of implicit schemes could provide damage assessment capabilities in individual fibers and their effect on the nonlinear response of the yarn.^[^ ^146^ ^]^ The influence of twist loads and filament geometries can also be investigated to understand the RVE geometry model failure mechanism under diverse loading scenarios.^[^ ^148^, ^150^ ^]^ These models can also be used to understand yarn softening as a function of twist per turn due to higher strains in the outer filaments than the core filaments. Composite structure and dynamic yarn‐fiber damage failure models must be perfected by incorporating nonlinear material models during material property definition in a MS simulation setup (see **Figure** **7**). Scalability and transitions in MS FEA modeling have yielded insights about composites’ strength dependency on twist and microscale constituent filament properties.^[^ ^147^ ^]^ As more diverse and multi‐phasic composites emerge, FEM could simulate composites or yarn (such as Kevlar) tenacity based on the constituent filament geometry and mechanical properties. Such models would be an exciting research proposition. This approach would help relate localized parameters (at micro‐/mesoscale) with homogenized (continuum‐/macroscale) properties and verify model fidelity with experimental results.^[^ ^149^ ^]^

MS model ensembles could be subjected to a series of loads (tensile, twists, or both). For example, variable twist load definition as a function of yarn radius can be defined as B.C. to analyze its impact on yarn strength. These FEMs will simulate composite strength behavior under various loading scenarios and aid in down selection by eliminating insignificant model cases and limiting the need to execute complex experimental tests physically. These research projects have helped optimize composites or yarn tenacity and evaluate their application in complex composite structures. The need for high fidelity and faster MS hard composite computational models is on the rise for analyzing diffusion in a metal matrix, radiation‐induced damage in ceramics, nanocrystalline alloy strength evaluation, crack propagation in brittle solids, polymer chain relaxation in nanocomposites and nucleation control rates analysis in biomimetic composites,^[^ ^59^ ^]^ etc.

### 2.4 Artificial Intelligence (AI)‐Assisted Modeling in Composites

AI (ML/deep learning [DL])‐based computational frameworks have emerged as powerful predictive tools for data‐driven multiphysical modeling, leading to unprecedented insights and explorations of the material properties beyond the capabilities of conventional numerical modeling.^[^ ^15^ ^]^ Most of the developments in ML/DL codes for composites are intended to approximate physics‐based modeling tools such as MD and FEM by acting as a surrogate and yield results at computational cost orders of magnitude less. ML codes explore the design space of composites^[^ ^151^ ^]^ rapidly and yield high‐performance composites (i.e., material discovery applications^[^ ^152^ ^]^). Chen et al.^[^ ^153^ ^]^ provided a succinct introduction to ML codes (linear regression, logistic regression, and neural networks). Their work toward hierarchical composite design inverse composite modeling predicted the need for efficient topology representation for composites, which gained much attention in the following years. Over the past two decades, coupled models (as analyzed in Section 1) have increased exponentially, incorporating nuances of purely computational methods, steadily integrating a data‐driven approach that increases model efficiency and makes complex physical models less strenuous. This section will summarize some of the most notable contributions of AI (ML/DL)‐assisted models for both hard and soft composite material families. As the ML landscape is fast evolving, several reviews and reports have come out recently about opportunities and impact of ML in materials: Cassola et al.^[^ ^154^ ^]^ in their recent review, discussed the impact of ML in composite material design processes and how data‐driven tools can be integrated into process simulations. ML‐based models have also been instrumental in composite materials (nonlinear) constitutive modeling. In a 2021 review from Liu et al.^[^ ^17^ ^]^ various use cases and opportunities of applying ANN models for data‐driven composite modeling, design, and analysis via physics incorporated (PINN) ML models to approximate elastoplastic, viscoplastic, hyperelastic, damage and traction‐separation behavior. On these lines, a 2019 paper from Liu et al.^[^ ^155^ ^]^ also proposed a deep material network (DMN) for MS topology in a nonlinear plasticity problem using topological representation of RVEs, DMN could yield high‐fidelity efficient concurrent simulations. This provided a mechanistic understanding of structure–property relations across composite length scales and aided in developing the parameterized microstructural database.

**Table** **8** summarizes the critical model contributions in studies concerning composites using the ML model. MS composite material research has successfully used different variants of ML algorithms and architectures for different purposes, such as predicting material properties, optimizing composites by analyzing stress–strain relationships, and generating composite structural mapping with their behavior under different conditions.

**Table 8:** Summary of ML‐based models for composites materials research

Material Model	Source of database	[ML] Model	Input	Output	Reference Number
Two‐phase periodic composite	FEM	ANN	Microscale stress at each point (voxel) of the RVE	Macroscale strain (applied as loading conditions) caused observed stresses	[320]
Microscale volume elements (MVE) were generated with 100 distinct VF values	FEM	Tree‐based model	57 voxel‐level features	Local response in spatial voxel	[321, 322]
MVE (MVE), two‐phase materials.	FEM	Linear multivariate regression	N‐point spatial correlations	1) Effective yield strength and 2) partitioning of imposed strains among microscale constituents	[323, 324]
3D MVE, two‐phase materials	FEM	CNN	51 × 51 × 51 3D microstructures, each voxel assigned a value of either zero (i.e., hard phase) or one (i.e., soft phase)	Elastic properties (Young's modulus)	[325, 326]
Trabecular‐level bone	Experimental and FEM	ANN	Set of bone material parameters, boundaryconditions and applied stress	Average density (ρ), average damage (D), average elastic modulus (E), and average stimulus (S)	[327]
Nonlinear anisotropic materials	FEM	ANN	Anisotropic strain data	Anisotropic stresses data	[328]
Two‐phase composite	FEM	GAN	5,000 synthetic microstructure images of size 128 × 128 are created using Gaussian random field method	the mapping between latent variables and microstructures of composite material	[329, 330]
Polyvinylchloride (PVC) composites	Experimental	ANN	Weight percentages of virgin PVC, recycled PVC, CaCO_3_, DOP, CPW, and CaCO_3_ particle size	Tensile strength, ductility, and density	[331]
Composite obtained by a mixture of hemp and polypropylene fibers	Experimental	ANN	Layer orientation and cutting direction	Tensile strength and elongation at break	[332]
Mechanistic homogenized RVEs (2D) and 3D RVEs as patterns of the two‐layer structures, high‐contrast composites modeling	Linear elastic RVE data (FEM), offline elastic datasets (DNS)	Deep material net (DMNs), SGD with back‐prop	2D‐RVE, plain strain condition ‐ high‐fidelity DNS and experiments. Bold‐driver algorithm for hyperparameter	Complex material responses, structure–property relationships (microstructures)	[155, 325, 333]
Isomap (nonlinear dimensionality reduction)	3D simulations of statistical RVEs (FE solver)	ROM (reduced order modeling) NN	Manifold‐based nonlinear ROM (MNROM) displacements (Mode 1 to 4)	Macroscopic loading parameters	[334]
Self‐consistent clustering analysis	Linear elastic simulations	Clustering (unsupervised ML) Data compression algorithms	Irreversible deformation (plasticity and damage	Small‐strain elastoplastic behavior, constitutive law for nonlinear composites	[335, 336]
Gradient‐boosted tree regression UD fiber composite	FEM, statistical volume elements	Data analytics and supervised ML	Image of material microstructure, CMs (loaded in the transverse plane)	Macroscopic stiffness, yield strength	[322, 337]
Tunable composite systems (2D)	FEM model	Linear (MLP classifier) and CNN	Displacement boundary conditions: FEM, varying VF, grid‐sized elements	Predict toughness and strength	[338, 339]
Composite and porous medium model for thermal properties	Numerical simulations (FEM) or experiments	Support vector regression, Gaussian process regression, and CNN, PINN	QSGS (to generate random porosity) and LB method	Effective thermal conductivities	[340, 341, 342]
Design hierarchical composite materials (Biomimicry)	Simulation, additive manufacturing, and experimental	CNN	Data matrix (enumerating unit cells in 1, 2, and 3's)	Generate optimized microstructure patterns	[343]
2D checkerboard composites	FE analysis. BVP and FE analysis	CNN, genetic algorithms, Monte Carlo, central value theorem (CVT)	FEM has displacement boundary condition	Stiffness, strength, and toughness	[344, 345]
Hierarchical and binary composites complex stress/strain modeling	FEM	Conditional generative adversarial neural network (cGAN)	Ensemble loading conditions	Material microstructure influence on physical fields like stress and strain	[339, 346, 347]
Graphene‐reinforced metal matrix composites	Hyperplanes from experimental sample points (phase content, hardness, tensile strength, heat treatment, ductility, and density)	ANN, K‐nearest neighbor (KNN), RF, SVM, and gradient boosting machine	Material and tribological variables as inputs: sliding distance, normal load, speed, counter face, and tribotesting method	Friction and wear modeling wear rate and coefficient of friction	[348, 349]
Brain (soft tissue modeling)	Experimental	3D CNN	Medical images	Brain lesion segmentation	[350]
Chemically treated collagenous Tissue	Experimental	CNN	Representative SHG image slices of a tissue sample	Two stress–strain curves (S11, E11) and (S22, E22)	[329]
Nanocomposites	Experimental	ANN	Matrix material, filler material, filler'sweight percentage, filler's diameter, and filler's aspect ratio	Young's modulus	[351]
Soft biological tissues	Experimental	SVM, bagged decision trees, ANN	Circumferential and longitudinal directions	Material parameters of the Gasser, Ogden, and Holzapfel strain energy function	[352]
Polycrystals	Experimental	ConvLSTM	External strain loads of oligo crystal and image of initial microstructure (orientation ﬁeld)	Stress evolution	[353]
Synthetic and authentic porous media images	Simulated data	CNN	Porosity and surface area ratio	Permeability	[354]
Particulate‐reinforced alloy composites	Experimental	ANN	Different particle sizes (mm)	Tensile strength, hardening behavior, and density properties	[355]
BWM	Experimental and FEM	3D‐CNN	3D voxel geometric information	Anisotropic material properties,	[356]
BWM	Experimental and FEM	SVM	Axonal fiber geometry information	fiber VFs	[357]
Soft tissue	Experimental and FEM	GP	Displacement, shear modulus	Material parameters	[358]

Table 8 briefly summarizes several successful attempts of the different ML/DL models applied in current studies of MS composite material (both hard and soft). As evident, the ML footprint in composites is swiftly increasing, and for the scope of this review, only a fraction of it has been captured by focusing on some of the most interesting and notable contributions. The authors would also like to stress that this combined take on the ML/DL model for the composites domain also encourages the transfer of ML across material families. The different input and output sources for the ML models explained the ML potential and data‐driven potential to solve variant MS composite material modeling problems. The ML methods varied from linear to nonlinear models, covering the ground areas of DL methods such as CNN, GAN, and long short‐term memory (LSTM). Based on the complexity of the modeling problem, main ML methods combined with reliable data sources present effectiveness and success in applications of MS composite modeling.

Deviations and variability in experimental results and input data (homogenized material property definition) significantly influence computational model efficacy (FEM or ML). ML has emerged as a prospective method to quantify these uncertainties. Such variability could be due to model architecture, sensitivity, or inherent variance/randomness in material property data. Sharma et al.^[^ ^15^ ^]^ discussed the role of ML in handling such uncertainties for composite modeling. Tang et al.^[^ ^156^ ^]^ proposed a MS uncertainty quantification method combining FEM, deep NNs, and sensitivity analysis to evaluate the tensile response of 3D angle‐interlock woven composites probabilistically. A two‐step numerical simulation creates the required dataset for training and validating the model. The role of each uncertainty in determining the macroscopic properties’ variance can then be established with fewer computational resources.

Similarly, Chahar et al.'s latest work proposed tackling this issue through multifidelity ML‐based surrogates, which can use a training dataset consisting of optimally distributed high‐ and low‐fidelity simulations.^[^ ^157^ ^]^ Suraj et al.^[^ ^158^ ^]^ proposed a ML‐assisted uncertainty quantification framework on unsymmetrical bistable laminates. As evident, the field of uncertainty quantification presents an exciting area of research, and there is tremendous scope for fast converging, cost‐effective, and reduced‐order techniques to reduce uncertainties in both soft and hard composites related to ML or ML + FEM hybrid models.

Discussion of AI in composites will be incomplete without touching upon large language models (LLMs) such as ChatGPT (OpenAI's Generative Pretrained Transformer (GPT)), Bing Chat, Google's LaMDA, and Bard frameworks being obvious candidates. LLMs have disrupted search engines and natural language processing space. There is tremendous potential in integrating these tools along with state‐of‐the‐art ML/DL models to create multimodal ML/DL frameworks that can solve even more complex composite process and damage mechanics problems by integrating (text, video, context) and currently used input parameters (RVEs, voxels, material data, etc.) to yield very high‐fidelity regression and classification deep net models in both hard and soft composites domain. Of course, cautious integration of LLMs with ML/DL nets would be necessary to ensure proprietary data is not compromised, and confidential computational codes will need due protection to safeguard industry stakeholders. It will be interesting to see how these next‐generation ML/DL + FEM + LLM + cloud‐based models will evolve in the next few years; for now, the sky is the limit!

## 3 Challenges in MS FEM

### 3.1 Material Model Definition: Soft Composites Focus

#### 3.1.1 Meshing

The challenge often with MS models is the need to tradeoff between model fidelity versus attainable model complexity, corresponding precision reduction, and increased output uncertainty. The introduction of efficient methods to distill information and transfer between scales, or the judicious use of fine‐scale information only in isolated parts of the domain while prescribing coarse‐grained formulations that couple to surrounding scale, is usually among the biggest hurdles.

Introducing finer meshing techniques that appropriately capture geometry helps increase computational accuracy in composite numerical models. Bas et al. proposed a higher‐order finite element known as the p‐FEM meshing technique for soft network composites to depict highly nonlinear deformation characteristics. This approach tends to increase the polynomial degree of the elements rather than the increasing number of elements to improve accuracy, cost‐effectivity, and speed in computing complex soft composite structures.^[^ ^159^ ^]^ Computational engineers must be wary of interpenetrations or gaps between elements when dealing with highly nonlinear material models. Cleaning up such microstructural discrepancies is a crucial preprocessing activity in many thermostructural simulations.^[^ ^160^ ^]^ A wide variety of mesh elements ranging from prismatic elements to tetrahedral and hexahedral elements (in modeling 3D fiber geometries) are available for modeling RVE geometries. Even though tetrahedral elements are easy to generate, they are not preferred in complex TBI modeling due to over‐stiffening and volume‐locking challenges, especially while modeling incompressible material models. Second‐order elements can be used instead to alleviate such difficulties, but this would lead to greater computation time and costs when compared to the hexahedral hybrid element type. Cheng et al.'s review summarized FE mesh elements, their benefits, application scenarios, and limitations for interested readers.^[^ ^41^ ^]^

Fiber geometries in soft tissues are typically modeled using lines or spline elements and must be converted into volume elements (voxels) before they can be integrated within a tissue matrix volume. Modeling the influence of such fiber orientations (axons/glia/other tissue fibers) in various sections of the continuum tissue geometry requires cautious meshing to avoid significant fiber overlap and obtain clean fiber mesh elements. Due to the paucity of experimental/histological studies, an exact number of fibers embedded in a region of interest (ROI) of soft tissue is difficult to estimate, and thus, defining proper fiber VF becomes challenging.^[^ ^161^ ^]^

Conforming hexahedral meshes and morphing approaches^[^ ^162^ ^]^ improve mesh transition at the interfaces between the cerebrospinal fluid and embedded WM fiber tracts. Subject‐specific head finite‐element geometry is developed via hierarchical imaging techniques. Another method that simplifies mesh generation while being computationally efficient is the feature‐based multiblock technique.^[^ ^163^ ^]^ Such regrouping techniques in BWM modeling can facilitate mesh morphing and generate accurate subject‐specific models. Lee et al. proposed a finite‐element strategy that generates adaptive mesh based on the degree of tissue anisotropy in WM. Their adaptive meshing scheme (named wMesh) relied on MRI structural information and fractional anisotropy maps from diffusion tensor imaging during the FE mesh generation step to optimally depict electrical properties in the human brain. Such meshing schemes improve the sensitivity and accuracy of FEM at interfaces of complex directional changes.^[^ ^164^ ^]^

#### 3.1.2 Computational Resources

MSMs are inherently computationally expensive, and there is often a need to judiciously estimate the extent of localized mechanical simulations necessary to make real predictions.^[^ ^41^ ^]^ Geometry simplification in finite‐element modeling (e.g., spring elements to depict reinforced fiber/pin geometries in laminated composites) is often required to reduce computational costs at the expense of model accuracy. Scoping out the region of interest (ROI) for precision modeling saves time and computing power. Similarly, arbitrary Lagrangian Eulerian‐based adaptive meshing techniques combining pure Lagrangian and Eulerian formulations have also been suggested to reduce computing costs.^[^ ^165^ ^]^ Researchers have also attempted homogenous equivalent modeling methods to disregard constituent phase differences that reduce computational workload, but such methods render localized studies impossible,^[^ ^4^ ^]^ thus highlighting the need for highly efficient graphics processing unit (GPU)/Message Passing Interface (MPI)‐based scalable simulation codes.

Optimization in MSM involves exercising multiple different models iteratively and is typically very expensive and intractable after a certain point. Although the advent of high‐performance computing (HPC) resources has tremendously enhanced computing speed, they are not enough to simulate several complex phenomena. In such cases, ML can help realize complex intensive models via surrogate or ROM. Data sampling techniques such as the Gaussian process (GP), Kriging (interpolation‐based method), etc. yield efficient predictions with lesser computational effort. Cutout techniques instead of iterative wrapper methods such as feature elimination or genetic algorithms (GA) can reduce computational workload.^[^ ^15^ ^]^ These new techniques can accelerate predictive modeling and material discovery‐related efforts. From a soft composite's perspective, these models have immense potential in tissue regeneration, aging, hybrid modeling, and characterization of complex physics involved in traumatic injuries use cases (e.g., blasts, crashes, fractures, and diffusional axonal injuries).

For current modeling approaches, tackling emerging problems involving coupling between atomistic or larger scales has been challenging to resolve with current computing resources.^[^ ^38^ ^]^ Spatial‐temporal coupling involving many transient physical timescales, such as diffusion or small‐scale deformation problems, has proven difficult to model and often needs large computing platforms to generate acceptable results. Hence, efficient scalable HPCs to compute faster solutions will become important.^[^ ^166^ ^]^ Such advanced numerical techniques facilitate the effective use of next‐gen supercomputers and help simulate large high‐resolution domains using accessible computing resources (such as commodity clusters). Devising modern parallel computing techniques will be instrumental in propelling computational materials engineering research utilizing high‐end supercomputers. Addressing MS simulation requirements is complex and requires an efficient, accurate information filter and transfer across scales and judicious use of fine‐scale information for formulations coupling to all the surrounding scales. To effectively utilize numerical tools, a cautious choice of simulation strategy guided by acknowledging current computational capabilities and multiplicative upscaling costs^[^ ^49^ ^]^ will ensure the best possible results. In the future, rapid and directed innovation in computing capabilities will help overcome some of the current modeling limitations.

### 3.2 Fidelity & Repeatability: MS FEM

Precise and realistic definitions of boundary conditions and material properties are crucial in determining model fidelity. Combining physics and data‐driven methods has proven very effective in synergizing high‐fidelity MS models at finer scales (micro‐ to the molecular level). Of course, fidelity comes at the cost of computational costs and complexities, but the modern approaches help formulate a much narrower design space. Combining data‐driven approaches aids in developing high‐fidelity models (HFM) in the early stages of materials processing, even with practically nonexistent experimental data.^[^ ^49^ ^]^ Once a working HFM is built, an extensive digital database is produced to create a low‐fidelity model (LFM) using ML/DL in a space constrained by conservation laws at coarser scale, boundary, initial conditions, and physics‐ or math‐based upscaling laws that may or may not employ scale‐separation principles. Fish et al. described such HFMs that can be further tuned in a MS framework for material discovery or design applications.^[^ ^49^ ^]^

Multifidelity learning architectures fusing data from disparate sources can build surrogates that outperform prediction outcomes based on a single data source. The trick is to establish correlations among accessible, inexpensive, low‐fidelity data acquired from simple computational/experimental models with slightly expensive, high‐fidelity data from more complex computational models or experiments. Fused, these models can then be deployed to make predictions for regions of interest (ROI) that lack high‐fidelity data using easily acquired low‐fidelity measurements.^[^ ^167^ ^]^ A novel mathematical framework such as materials knowledge systems has been recently formulated to extract, store, and recall computationally efficient hierarchical linkages at the core of MSM materials phenomena.^[^ ^168^ ^]^ These linkages can be used successfully to accurately predict the continuous evolution of microstructure over prolonged periods with high reproducibility.

MS simulations utilized for material property prediction must be validated against experimental observations. Furthermore, verification of the numerical scheme, sensitivity analysis to identify the most influential input parameters, and quantification of uncertainties in the input and output are essential to develop accurate simulation schemes. This methodology of validation, verification, and uncertainty analysis is called VVUQ. VVUQ ensures MS model transferability and scope of applicability by highlighting any erroneous assumptions.^[^ ^169^ ^]^

Ye et al. proposed a high‐fidelity generalized method of cells‐based approach to create microscopic models of stress distributions in the RVE. This MS method investigates composites’ failure behavior and damage evolution with reinforced fibers. At the part scale, integration points in each element are deployed to investigate damage evolution.^[^ ^170^ ^]^ Using a mechanics‐based ROM, a multifidelity ROM for MS damage analysis has helped accelerate microscale elastoplastic deformation by clustering the degrees of freedom. ROMs with well‐tuned damaged parameters can generate material response databases correlating microstructure morphologies with material behaviors under complex loading scenarios.^[^ ^171^ ^]^ Accounting for process‐induced uncertainties propagating across length and timescales is vital for robust design and analysis of composite structures.^[^ ^171^ ^]^

## 4 Outlook: Multiscale Composite Modeling

MS models for composites have evolved tremendously over the past two decades, and the increase in computing capabilities has played a significant role. Sequential modeling or bridging techniques in numerical models have made calculations accessible from one scale to another. MSM has propelled innovation in novel semiconductor materials (for instance, embedded‐capacitance composites for electrical circuit boards) and optimization of new nanocomposites that have revolutionized power transformation technology. Similarly, a wide range of flexible polymers used in electronic packaging have been products of advanced numerical solutions, MD or mesoscale models.

In the future, MSMs that can model large‐scale fractures and capture long‐term damage evolution in composites with high fidelity will be an exciting proposition for materials scientists. Stitching MS models helps in easy transformations from particle‐level to continuum‐level models and simulates large‐scale failure in granular or monolithic systems.^[^ ^59^ ^]^ Predictive worst‐case scenarios in MD simulations will yield new insights into atomistic failure in nanocrystalline composite materials and open up possibilities to study long‐timescale relaxations for composite systems containing full molecular detail.^[^ ^59^ ^]^ Physics‐driven and ML‐driven multiphysical models are needed to model complex composite material behaviors, especially for elevated temperatures or extreme operating conditions.

Similarly, while modeling soft tissue composite materials for the BWM, there is an opportunity to build connected models that can depict TBIs, aging, impact, concussions, and fatigue behavior. Generating volumes of data is critical for improving the fidelity of these models. In this regard, state‐of‐the‐art sensors such as accelerometers embedded in the headgear have been proposed to constantly sample accelerations experienced during high‐risk physical activities (such as those faced by athletes in contact sports).^[^ ^172^ ^]^ This data could be instrumental in developing data‐driven hybrid models to understand TBI and help advance research to prevent concussions. In this section, we will touch upon some up‐and‐coming research areas that could enhance the efficacy of results from computational models of soft and hard composites.

In Section 2.3.4., Table 6, phase field models were briefly touched upon. The prime characteristic of phase field models is the diffuseness of the interface between two phases, commonly called diffuse interface models.^[^ ^173^ ^]^ Phase field models provide tools for microstructure evolution analysis at mesoscopic scales. They rely on the thermodynamics of nonequilibrium states and find special applications in understanding transitions between multiple phases in multicomponent materials (hard and soft composites). AI‐assisted hybrid phase modeling could be an exciting avenue to explore and learn complex microstructure evolution dynamics in composites meant for high‐temperature or extreme environments applications. In the future, advanced phase field models, including diffusion and advection physics in multiphysical simulations (i.e., involving mechanical, electrical, and magnetic interactions), could unlock novel opportunities in microstructure analysis and design of engineered materials.

### 4.1 Meshless FEM

The FEM is widely used in developing computational biomechanical models. However, FE models suffer disadvantages when modeling soft materials such as tissues. Problems such as surgery simulation, blast loading of tissue, etc. are typically characterized by large deformations, which result in excessive element distortions that can lead to severe convergence issues. One way to overcome the issue is to employ an adaptive element remeshing technique as the simulation progresses, but this can be computationally expensive. Also, modeling the material and geometry across multiple length scales requires adequately capturing nonlinear material behavior and complex geometry. This is not easy to achieve accurately using traditional meshing techniques and is time‐consuming.

An alternative to overcome these issues is to jettison the idea of meshing and solving the equations of motion at each node based on its relationship with its neighbors. Several such methods are broadly classified as meshless methods.^[^ ^174^ ^]^ Miller et al. used the total Lagrangian explicit dynamics to compute patient‐specific deformations in the brain during surgery. The brain's anatomy is modeled using nodes whose relationship with its neighborhood is interpolated by a moving least squares method using quartic spline weight functions.^[^ ^175^ ^]^ Joldes et al. developed a 2D model of soft tissue under compression and extension using the element‐free Galerkin method by imposing essential boundary conditions (EBC). The section briefly explores two methods, one often used in modeling tissues, while the other is a new methodology with much scope for developing efficient, accurate models of tissues. A comprehensive review of meshless methods can be found in ref. [174].

A meshless method often used is the smoothed particle hydrodynamics (SPH), a mesh‐free Lagrangian method to obtain numerical solutions for differential equations combined with a CDM to predict tissue behavior. The mathematical implementation is achieved by explicitly solving the equilibrium equations in the referential form.

(24) $$\begin{array}{c} \nabla \times P+\rho b=\rho_{0}\ddot{x} \end{array}$$

**Figure 8:**
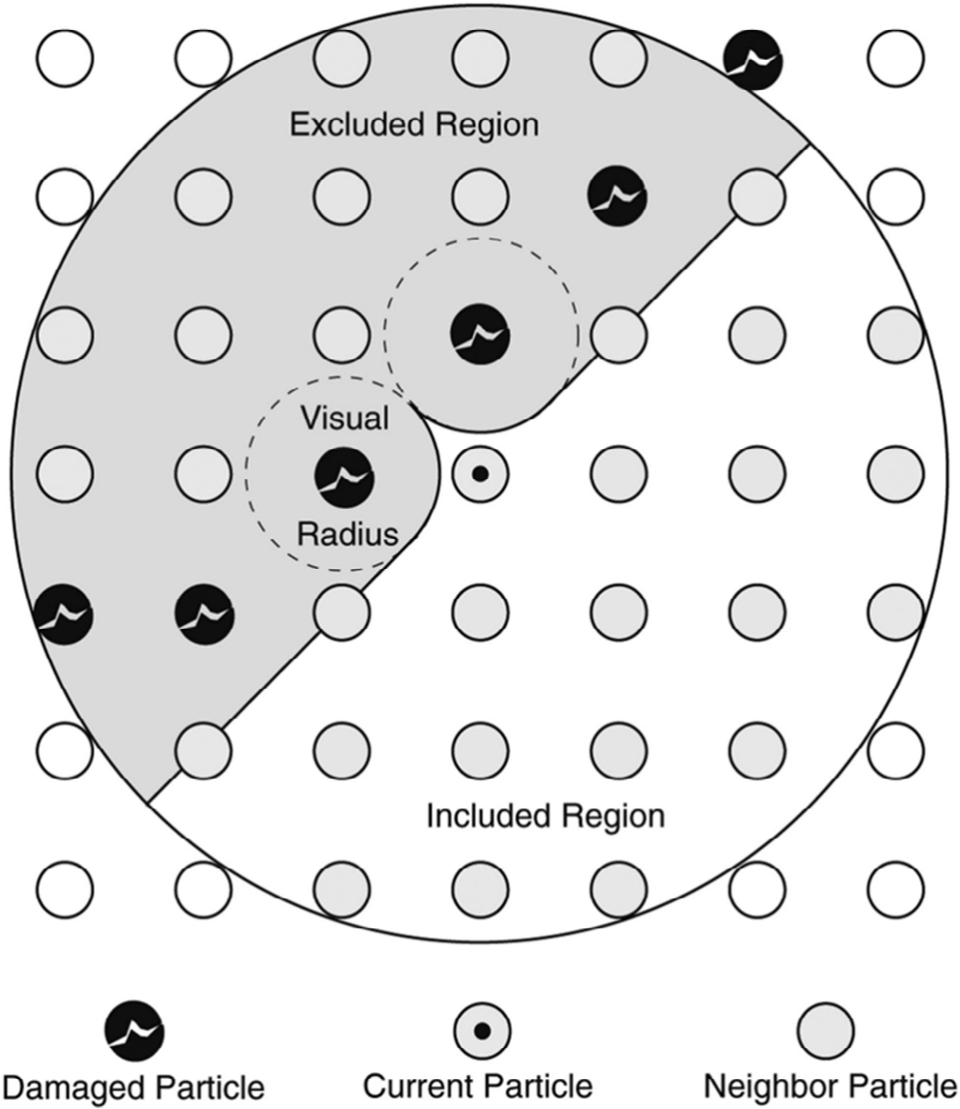
Discontinuous SPH damage and failure algorithm. The damaged particles are removed from the original list of particles. Reproduced with permission.^[^ ^176^ ^]^ Copyright 2016, Springer‐Verlag Berlin Heidelberg.

**P** is the first Piola–Kirchhoff stress tensor, **b** is the body force vector, **ρ**_**0**_ is the referential mass density, and $\ddot{x}$ is the acceleration. The strong local form of the equilibrium equation can be written as^[^ ^3^ ^]^

(25) $$\begin{array}{c} m_{i}{\ddot{x}}_{i}=f_{i}^{i}+f_{i}^{e} \end{array}$$

where $f_{i}^{i}$ is the internal force and $f_{i}^{e}$ is the external force on particle *i*. For any strain energy function, a damage model can now be introduced as a function of the load history using a stretch‐based continuum damage approach. The strain energy potential can thus be modified as a function of damage variable D.^[^ ^176^ ^]^

(26) $$\begin{array}{c} W(C,M,D)=(1-D)W_{0}(C,M) \end{array}$$

where *W* is the updated strain energy potential, *W*_0_ is the original hyperelastic strain energy function, and M is the referential fiber orientation vector. The second Piola–Kirchhoff stress and evolution of damage can be determined as^[^ ^176^ ^]^

(27) $$\begin{array}{c} S=2(1-D)\frac{\partial W_{0}}{\partial C},-\frac{\partial w}{\partial D}\dot{D}\geq 0 \end{array}$$

where *D* is a function of the model parameters, such as the applied stretch, the critical stretch, and other material parameters; in meshfree methods, the connection between particles is determined by the interaction between the neighboring list of particles at the beginning of the simulation. Modification of the list can create discontinuities in the model. It can be handled by checking for damage at each particle and updating the original initial list.^[^ ^177^ ^]^ Lluch et al.^[^ ^178^ ^]^ developed a total Lagrangian formulation using a corrected SPH to simulate cardiac mechanics of passive dilation and active contraction in an ellipsoidal left ventricle using an exponential anisotropic constitutive law of Guccione following the direction of cardiac fibers (**Figure** **8**).

Another method looks at a novel approach to the formulation of continuum mechanics and is gaining traction in modeling problems related to damage, crack creation, and propagation called the peridynamic theory. Peridynamics is a promising tool for modeling biological materials on the cellular and histological scales because the theory can capture nonlocal forces inherent in many biological systems.^[^ ^179^ ^]^ As is the case with several meshless methods, peridynamic models are comparably more expensive than traditional FEM models. However, peridynamic codes can be implemented within massively parallel processing frameworks that scale extremely well with several processors.^[^ ^180^, ^181^ ^]^ Furthermore, without the mesh requirements, we eliminate both expensive manual postprocessing steps required to model complex structures and simulation errors due to mesh failure. Large deformations can also lead to element distortion, requiring an adaptive mesh refinement algorithm.

Peridynamic theory is a nonlocal method that employs spatial integral equations instead of differential equations. As a result, the equations remain equally valid at points, surfaces, or discontinuities. Material damage also becomes part of the constitutive laws that permit fracture initiation and propagation.^[^ ^182^, ^183^ ^]^ For a body in a reference configuration *ℛ*, let each pair of particles interact through a vector‐valued f such that the force per unit reference volume due to the interaction $L_{u}$ is defined as the functional of the displacement field u. The value of $L_{u}$ at any reference point × at any time t is given by^[^ ^182^, ^183^ ^]^

(28) $$\begin{array}{c} L_{u}=\int_{ℛ}f(u^{\prime }-u,x^{\prime }-x)dV_{x},\;\forall x\in R \end{array}$$

where $x^{\prime }$ and $u^{\prime }$ correspond to the displacement field and position of the neighboring particle. The peridynamic equation of motion is given by

(29) $$\begin{array}{c} \rho \ddot{u}=L_{u}+bonR,\;t\geq 0 \end{array}$$

**Figure 9:**
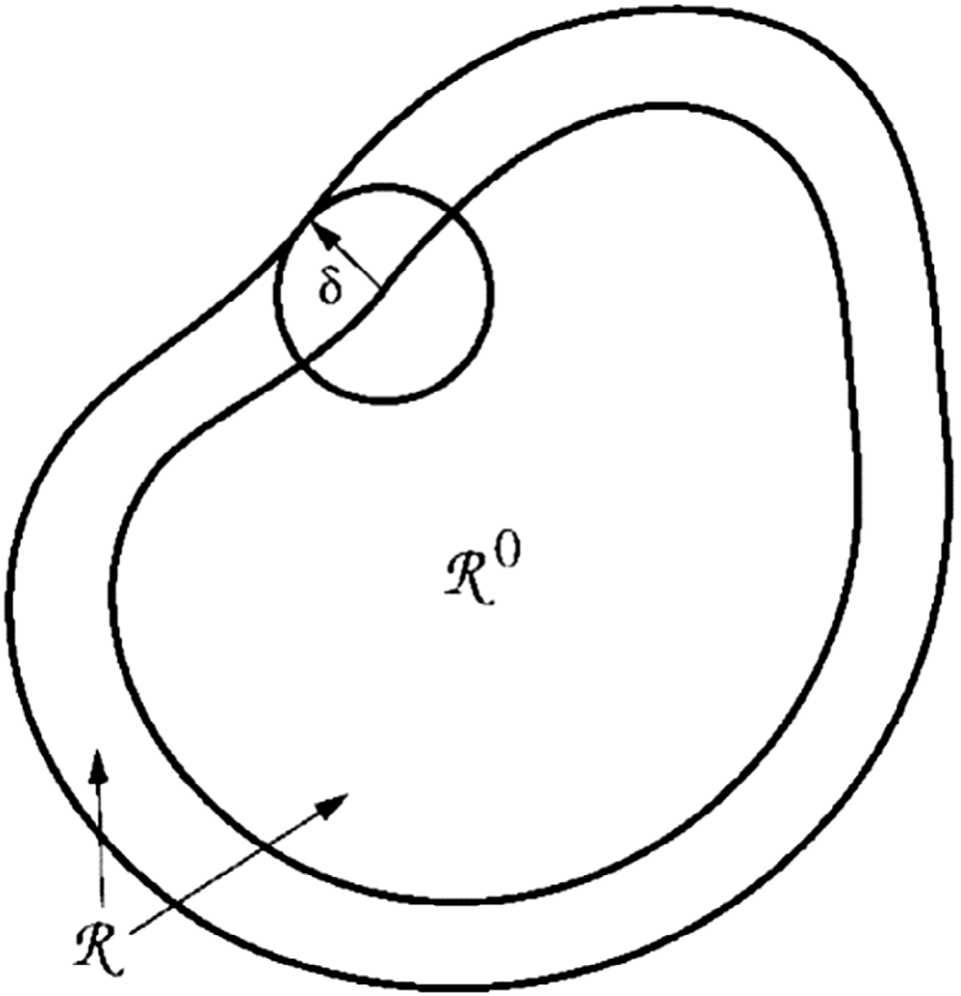
Representation of an internal subregion.^[^ ^183^ ^]^ Copyright 2008, Elsevier B.V.

Moreover, the equilibrium equation then becomes

(30) $$\begin{array}{c} L_{u}+b=0onR,\;t\geq 0 \end{array}$$

where b is the prescribed body force, and f is the pairwise forcing function. We can construct a form of f using the conservation of angular momentum and by restrictions imposed by Newton's law where the force on some particle 1 due to its neighbor particle 2 is equal and opposite to the force on particle 2 due to particle 1. By defining a horizon *δ*,^[^ ^182^, ^183^ ^]^ any particle within this distance from any other particle can interact, but particles at a greater distance do not interact. We can think of all particles within the region *δ* as belonging to ${ℛ}^{0}$ which is a subset of ℛ, as shown in **Figure** **9**. A notion of microelastic energy density functional W_u_(x) at any point × can now be defined using the form of the vector‐valued pairwise forcing function, **f**.

Lejeune et al.^[^ ^184^ ^]^ developed a computational model for tumor growth that can represent individual cells or whole tissue using the peridynamic theory. The model demonstrates a computational framework for cell division, an essential factor in tumor growth. Xu et al.^[^ ^185^ ^]^ demonstrated a peridynamic framework to regenerate hyperelastic materials such as St. Venant–Kirchhoff and neo‐Hookean.

Silling and Bobaru^[^ ^183^, ^186^ ^]^ formulated an antishear plane problem for an isotropic, microelastic, peridynamic body for asymptotic mode‐III crack tip, and the results were compared with the conventional theory. An interesting feature observed in the results is the cusp‐like shape of the crack with peridynamic theory. The result from conventional theories shows a parabolic profile for crack propagation, which requires infinite stress (singularity in linear fracture mechanics). Any real material cannot sustain infinite stress and requires modifications and special treatments for such problems in classical theory. Such modifications produce a similar cusp‐like feature observed as a natural outcome of peridynamic theory. Another application involves the examination of the theory for membrane with a mode‐I type slit. The model predicts wrinkles’ appearance along with the crack's propagation leading to fracture. The limiting crack growth velocity is well under the Rayleigh wave speed for the given material properties. The general features of the propagating crack agree well with experimental results. Physically impossible strains which plague classical theories do not occur in the peridynamic result. Huang et al.^[^ ^187^ ^]^ developed an FE‐based peridynamic model using the Abaqus FE software. The researchers developed user‐defined element Fortran subroutines to implement the peridynamic theory in Abaqus. Kilic and Madenci^[^ ^188^ ^]^ developed a coupled FEM peridynamics model that exploits the advantages of both FEM and PD approaches, that is, fast numerical efficiency and inherent crack prediction capabilities, respectively.

The peridynamics approach has been employed only for domain portions where damage was expected to occur, whereas the FEM approach has been employed for zones where damage is not expected. Lehoucq et al.^[^ ^189^ ^]^ implemented peridynamics within an MD framework, thus enabling mesoscale and macroscale modeling of materials. The force interactions that result from discretizing peridynamics are similar to the traditional forces seen in MD. As a result, peridynamics can be easily implemented with minor modifications within an MD code. However, peridynamics is a continuum theory; therefore, individual atoms need not be modeled, and a true physically correct interatomic potential need not be known to use peridynamics within an MD framework effectively. The model is implemented in LAMMPS MD software for an impact simulation of a rigid sphere on a homogeneous brittle material. The results are compared with FE simulations, and they agree qualitatively with FEM, in size, shape, and the distributions of resulting fragments.

While meshless methods have several advantages over FEMs, they have certain drawbacks. EBC are often not easy to enforce. Meshless methods can be computationally expensive as many Gauss points may be required to solve the weak‐form equations.

### 4.2 Hybrid FEM

Data‐driven ML codes are becoming increasingly popular in multiphasic polymer composite materials design and analysis. Such approaches combining ML and physics‐based MS FE simulations are expected to accelerate material discovery and informatics. Typically, FE HFM are used to construct LFM using ML) within the constraints determined by constitutive laws, boundary and initial conditions, and any other relevant physics‐ or math‐based upscaling laws that may or may not employ scale‐separation rules. Once the surrogate LFM is trained, the HFM can be coarsened and tuned for material discovery or design applications.^[^ ^49^ ^]^ The use of ANN in the role of the constitutive operator is not a new concept. The classical application of ANN in the constitutive modeling of concrete was initially proposed by Ghaboussi et al. in 1991.^[^ ^190^ ^]^ Similarly, ML codes were integrated as part of FE code, as discussed in research proposed by Gawin et al. in 2001.^[^ ^191^ ^]^ Shin and Pande^[^ ^192^, ^193^ ^]^ also proposed hybrid FE‐ANN codes whereby they showed that inserting the constitutive laws presented as a neural operator led to qualitative improvement in the FE model outcomes.

A similar hybrid FE/MD/TB simulation framework has been proposed to compute forces on particles and consequently derive particle positions and velocities in a time‐stepping algorithm.^[^ ^38^ ^]^ The inspiration behind incorporating ANNs into different branches of engineering stems from analyzing the transmission and transformation of signals in the human brain (neurons). Research on hybrid FE‐ML models for soft tissue (BWM) characterization has gained much traction. Recently, Wu et al. developed a homegrown hybrid FE‐ML data‐driven technique to predict the mechanical response of WM at various locations when subjected to frequency‐dependent shear strains. Such hybrid algorithms are finally intended to result in a CNN model that predicts the anisotropic mechanical properties of specific ROIs of the central nervous system. The hybrid model of 3D‐ResNet with 3D anisotropic REVs successfully overcame limitations encountered in traditional FEMs, such as high computational cost and complicated geometrical mesh failure problems,^[^ ^194^ ^]^ and served as an efficient surrogate to predict anisotropic material properties.

ML‐assisted hybrid models have also been deployed for hidden feature identification, such as spotting undiscovered features that could affect the global composite material response. This is particularly useful for complex material and microstructural composite material systems.^[^ ^15^ ^]^ The data science‐driven approach in hybrid models preprocesses training data (e.g., PCA) to reduce the input dimensionality, thus significantly reducing computational costs. However, one must overcome challenges in connecting different elements into a single system so that ANN models do not lose information across different elements.^[^ ^17^ ^]^ Hybrid models are also a promising avenue for developing new bioinspired composite materials while addressing various inverse design problems.^[^ ^153^, ^195^ ^]^

Further breakthroughs in ML‐FEM models for composite materials can be expected by developing more efficient topology optimization codes for composites and inverse design methodologies.^[^ ^153^ ^]^ Another practical hybrid model application is data‐driven hybrid FEM‐ML surrogate models tasked to predict composites’ interface bond strength quality and determine their dependence on the process settings. The model accuracy is validated with experimental cross‐tension test data.^[^ ^196^ ^]^ Well‐defined, efficient, and scalable hybrid models are the foundation for developing DTs and cyberphysical production systems (CPPS) for composite materials discussed in the following section.

### 4.3 DT‐Material Model

DTs are computer‐generated digital equivalents of physical systems. They simulate various key performance objectives through real‐time harmonization of data received from experimental/manufacturing facilities.^[^ ^197^ ^]^ The fidelity and interpretation of DTs are closely related to the simulation techniques and software used.^[^ ^134^ ^]^ For composite modeling, DTs could be an innovative approach for modeling, optimizing, and simulating soft and hard composites. DTs are essential for developing cost‐effective, precision, and safety‐critical applications where robust material manufacturing is needed. DTs are touted to significantly improve manufacturing time, mechanical performance, and weight. An exciting area of research under Industry 4.0, known as CPPS, is being built to set up DTs by combining ML and FEM.^[^ ^196^, ^198^ ^]^

In a DT, the parametric FEM component generates the training data for the ML component, which helps build the FEM surrogate models. Data is the currency that enables information transactions in a DT. Different data‐driven methods are being experimented with to build materials processing, synthesis, characterization, and supply‐chain twin systems. The key aim of DTs would be to yield optimal process conditions and reduce experimental testing and run‐in processes. Materials DTs are made of many digital threads that identify and control critical process variables in the composites or advanced materials manufacturing processes in real time. DT at the enterprise level is built on data‐driven approaches. These digital threads are deep simulation models that are computationally expensive and very difficult to execute close to real time without noticeable latency. Hence, surrogate models are implemented to search for large parameter spaces efficiently. These surrogate models adequately represent the physical processes and systems within milliseconds’ latencies.

Composite structures conventionally fail due to damage accumulations and environmental interactions. Effects on their structural performance are often determined by emergent behavior described by degradation events and external factors.^[^ ^199^, ^200^ ^]^ Accurate DT models can improve material prognosis by improving damage prediction. For instance, DT models based on the “filling information gaps” technique for the propagation of crack paths have been used to help predict crack paths in composite structures.^[^ ^201^ ^]^ Digital representation of such fiber manufacturing processes can offer real‐time fiber adaptation capabilities. Similarly, real‐time simulation models can predict and define manipulated fabric topologies.

Surrogate models can perform fast and exact searches within the parameter space, making them suitable for inline quality control applications for composite material processing systems. Of course, these models must be backed with proper sensor‐generated data to yield reliable process simulations and recommendations. DT is based on comprehensive parametric models that serve as ML input to reach high‐fidelity FEM surrogate models for soft and hard composites. As technology in hardware improves, computational time costs will continue to decrease. Encoded surrogate simulation platforms will enable a secure real‐time configuration of actual process variables that evaluate real process data observations and thus improve overall composite manufacturing and structure performance.^[^ ^134^ ^]^

### 4.4 State‐of‐The‐Art Material Model Definition: Soft Composites

Future possibilities in enhancing current computational models are immense. A unified numerical modeling solution that is easily scalable in length and time would be the next big step in computing. Such models should be parametrically controlled to define complex physical boundary conditions and then backed by a data‐driven paradigm to reproduce highly accurate and repeatable predictive properties. Such MS and multiphysics composite models would be a giant leap in advancing our understanding of material performance, damage, and aging properties across scales ranging from quantum to macroscales. In the semiconductor industry, thermal buildup is an active research area, focusing on developing reliable electronics. This presents opportunities to generate new thermoelectrical performance simulation codes to improve material failure prognoses.^[^ ^4^ ^]^

Similarly, for hard polymer composites, modeling the effect of curing needs to be improved, and this would provide insight into interfacial dynamics in complex laminated structures and woven fibers/yarns. Sound theoretical models will continue to be the backbone for developing reliable material simulation codes. For instance, linear–elastic and hyperelastic CMs will continue to be used to depict rubber‐like materials, but the application dictates the choice of model. Viscohyperelastic models will be used to characterize the mechanical behavior of elastomers.

**Figure 10:**
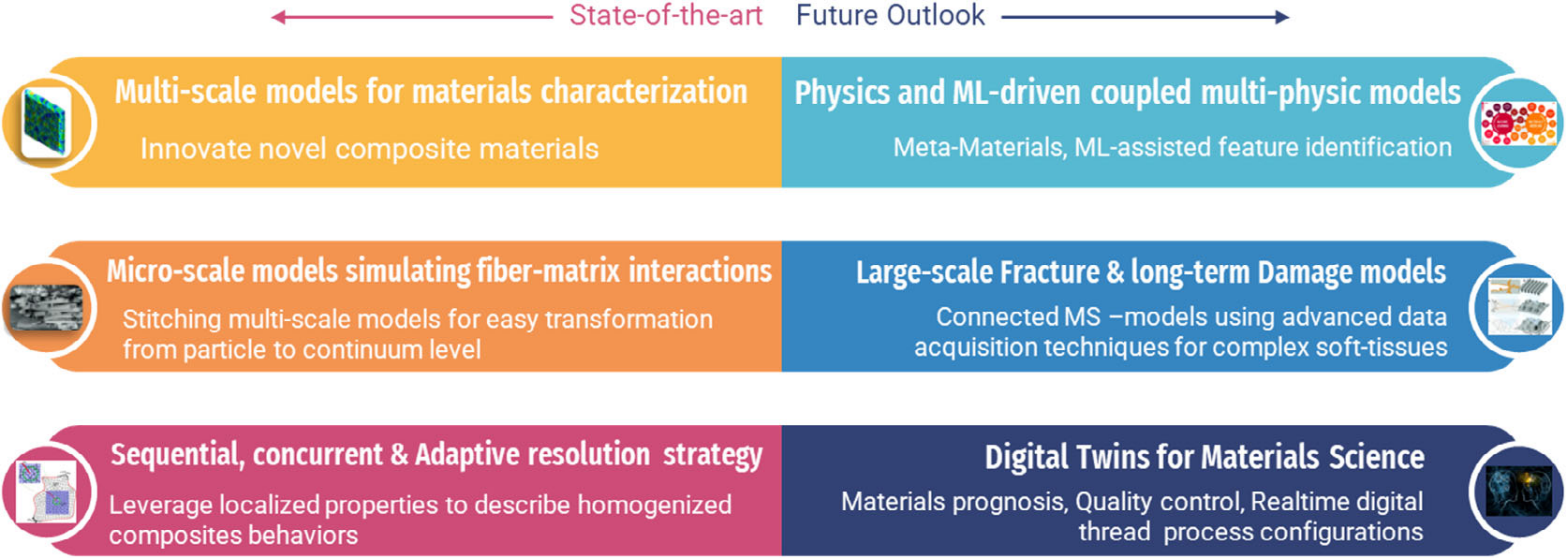
State of the art in composite materials modeling ranging from material characterization and fiber–matrix interactions via sequential, concurrent, or adaptive resolution strategy. In the future, MS models will have massive scope in large‐scale fracture long‐term damage mechanics modeling and developing DT solutions for materials science.

Reliable and highly mimetic damage and fatigue models are another hot topic of interest for soft tissues in BWM to understand material decay characteristics. Mathematically, such phenomena for composites would use strain or energy density equations, as discussed previously. Researchers have put forward systematic coarse‐graining and back‐mapping schemes as avenues to couple MS models and leverage local properties to describe homogenized behaviors. Domain coupling is another up‐and‐coming solution method under a sequential model schema. The concurrent models’ approach is desirable, and there is keen interest in simulating complex metal and carbon nanocomposites. Finally, the adaptive resolution modeling method would simulate various spatial and molecular resolutions (**Figure** **10**).

From a FEM standpoint, the application of SEM (spectral element technique) has improved the precision of FEM by combining spectral techniques with finite‐element codes. This method is an up‐and‐coming solution to solve viscoelastic polymer composite flow mechanics. Such models can inspire merging applications among other computational models, too. They leverage the best individual solutions to design a superior computational package. Such improved methods could better predict phenomena such as curing, polymer flow in dilute and concentrated solutions, polymer layer characteristics close to nanoparticle surfaces, molecular roots of viscoelasticity in filled elastomers, and dynamics of confined polymers.^[^ ^38^ ^]^

The discovery of new CMs for nonlinear composite phenomena, such as the peridynamic theory, could help solve some of the existing computational challenges. It will be a good test of the current computational tools and help validate such mathematical models. The research community strongly emphasizes improving, benchmarking, and executing state‐of‐the‐art models by validating transferable model parameters and refining MS model interfaces.

### 4.5 Material Aging, Damage, and Evolution

Damage in soft tissue can be described as the deterioration in the mechanical and functional performance of the tissue due to factors such as large loads, large deformations, and repeated loads. Damage may occur due to a combination of the above factors or deterioration of the material properties due to disease and aging. Damage modeling encompasses several scenarios, such as stress softening or Mullin's effect, strain softening under large strains, damage accumulation, and tissue rupture encountered during trauma‐like events and surgeries. Damage models are useful tools that shed light on tissues’ phenomenological behavior and damage progression over time. Simo^[^ ^202^ ^]^ developed a 3D nonlinear viscoelastic model with an isotropic damage mechanism within the framework of continuum damage mechanics. Damage is characterized by the maximum value of strain energy previously attained by the undamaged material under loading. The damage variable *D* is the conjugate variable to the strain energy density function and is characterized by an irreversible damage evolution equation. Rausch et al.^[^ ^176^ ^]^ developed a smooth particle dynamics (SPH) approach using the above damage formulation in modeling soft tissues. Volokh^[^ ^203^ ^]^ formulated a softening hyperelastic modeling approach by extending the fracture mechanics analogy to hard materials. It is achieved by introducing an expression for the critical failure energy, indicating the maximum strain energy sustained by an infinitesimal element before material failure. Holzapfel and Ogden^[^ ^89^ ^]^ proposed a mechanical model that accounts for damage accumulation in collagen fibers under shear loads. The model is analogous to Mullin's effect. Begonia et al.^[^ ^204^ ^]^ performed quasistatic stress–strain tests on porcine brain tissue and developed a CDM under mild TBI loading. Cheng et al.^[^ ^205^ ^]^ developed FE models that predict tissue damage severity during robotic‐assisted minimally invasive surgery.

Sack et al.^[^ ^206^ ^]^ investigated the impact of aging on the material properties of brain tissue by fitting multifrequency MRE measurements to different rheological models. The study was based on fitting a spring‐pot viscoelastic model to frequency‐based MRE measurements. The study predicted a linear trend in the decrease of the stiffness parameter of brain tissue with age. Flynn and McCormack^[^ ^207^ ^]^ developed a finite‐element model of the formation of wrinkles on the skin due to aging. The three‐layer model of the stratum corneum, dermis, and hypodermis combined Prony series parameters in the time domain and a hyperelastic strain energy density function for each layer. Diosa et al.^[^ ^208^ ^]^ formulated an FE model of a dynamic indentation test on the skin to predict the effect of changes in microscale topography due to aging. Antona–Makoshi et al.^[^ ^209^ ^]^ developed an FE model to study the relevant factors for the thoracic fragility of the elderly in frontal impact scenarios. Acun et al.^[^ ^210^ ^]^ performed in vitro experiments and developed human‐induced pluripotent stem cell (hiPSC)‐derived cardiomyocyte‐based aged myocardial tissue as an alternative research platform to study cardiovascular disease progression. Damage modeling in soft biological tissues is still in its nascent stages. While several developments have been achieved in this area, the ability to predict damage in human tissues, specifically the brain, is still in its preliminary stages. Developing accurate MS models that are informed by experiments can lead to improved understanding of injury biomechanics and providing preventive care.

## 5 Conclusion

In this article, an in‐depth review of MS models for composite materials has been provided. The category of composites chosen was based on how closely they aligned with work done by the AMSL group at Rutgers University. This effort summarized related research on hard composites (polymers, fibers, and nanocomposites) and soft‐tissue composites (primarily BWM tissue). Various models have been discussed, ranging from molecular‐scale (atomistic simulations), micro‐, and mesoscale (FEM, CFD, multiphysics models) to ML/data science‐based reduced‐order surrogate models. The readers from the computational materials community would benefit from this article by obtaining a bird's eye view of the computational research landscape for composites. This research also explained futuristic computational solutions such as hybrid modeling and DTs, which could propel next‐generation real‐time computational model development with high fidelity and lower latency.

Investment in computational research, scientific training development of computational engineers, and improvement in computational platforms have contributed to value generation from numerical modeling in materials science. As elaborated previously, MSMs have enabled the simulation of microscopic structures and depict interactions/bridging mechanisms across time and length scales (Section 2). For modeling connections between the fibers and the matrix, deploying approximate solutions such as connector elements like linear and nonlinear spring or spring‐dashpot connections becomes necessary, as outlined in discussions from research by the AMSL group at Rutgers.^[^ ^73^, ^74^ ^]^ Improving fidelity in such micromechanical models would pave the way for a standardized approach to model soft tissues.^[^ ^41^ ^]^

With the rise of hybrid modeling, manufacturing considerations within the numerical model workflow have become pertinent to understanding curing mechanisms and parametric analysis of composite structures. However, further research is required to model such geometries at a large scale to yield robust and repeatable prognoses. MSM researchers could adopt sequential, adaptive resolution, or contemporary modeling approaches.^[^ ^38^ ^]^ Sequential models have proven very effective in predicting material behavior, while concurrent models help link models across scales, and localized properties can be leveraged to feed into macroscale models to obtain properties across scales. Intricate statistical sampling techniques, such as the multicanonical and Wang–Landau methods, effectively simulate large composite systems’ phase behaviors. However, concurrent MS models are complicated and more computationally expensive than sequential approaches, particularly for solving material flow problems. MSMs are still poorly developed to characterize complex polymer flow physics in dilute and concentrated composites, interactions between heterogenous polymer–nanoparticles in PNCs, viscoelasticity behavior in filled elastomers, dynamics of confined polymers composites, etc. Hence, more effective concurrent models need to be developed.^[^ ^38^ ^]^

As a future objective, computational models focused on simulating different assembly patterns and their influence on hard and soft nanocomposite materials can be further explored. Linking FEM and ML models by hybrid, MS surrogate, or DT models can help investigate composite structures from multiple perspectives. Data analytics and physics‐driven ML models would be essential to obtain fast and affordable analysis to model complex failure initiation and damage mechanisms. Hence, maintaining a digital thread of all manufacturing steps would be essential to generate valuable data currency to develop surrogate ML models (Section 4). Damage initiation, propagation, and prevention modeling is a field that still needs much exploration. Mathematical models that could work for various scenarios merit further research for soft and hard composite structures. A nucleation study via accelerated dynamics technique has been put forward to understand nucleation and crack growth self‐assembly of synthetic hierarchical composite materials. Developing universal frameworks that can transform from particle to continuum‐based representations will facilitate easy large‐scale simulations of failure in complex nanocomposites. Including worst‐case scenarios in dynamical models will yield tremendous insights into nonlinear atomistic to continuum‐scale failure dynamics in nanocomposite crystal structures.

Efficient MSMs could lead to faster material discovery by enabling the design of experiment analysis simultaneously on many scales instead of performing slow and expensive experimental iterations. Domain coupling is a significant problem in MSM, and there is plenty of scope to synergize a generalized framework that can enable effective handshaking between domains. It would also be a landmark solution to develop a one‐size‐fits‐all computational code that can be deployed to assess molecular‐/micro‐/meso‐, and macro‐scale problems within one package that yields high fidelity, end‐to‐end effective, single‐interface computational solution. In this vein, the authors posit that there are opportunities to implement DTs for conventional material processing and synthesis. Equipment can be configured to generate material processing data, and a digital thread for the in‐house composite manufacturing process in a lab setting could be attained. Next, it is coupled with ML and FEM models to set up a DT model for hard composites to control critical process parameters and consequent part properties. It is a strong recommendation from the author that material researchers leverage such data‐driven surrogate models to simulate complex processes and, at the same time, control resultant composite part characteristics in real time, that is, Industry 4.0 material manufacturing philosophy.

Computational researchers should continue pursuing the overarching goal of realizing models that can enhance understanding of complex physical processes and control mechanics. This is especially necessary for complicated soft tissues (such as brain matter) that are difficult to characterize in vitro and validate against computational counterparts. While these challenges are daunting and some beyond current computational capabilities, innovations in computational hardware and advanced algorithms present a promising future for material scientists in their motive to attain real‐time high‐fidelity numerical composite simulations.

## Conflict of Interest

The authors declare no conflict of interest.
